# Human Immune System Diseasome Networks and Female Oviductal Microenvironment: New Horizons to be Discovered

**DOI:** 10.3389/fgene.2021.795123

**Published:** 2022-01-27

**Authors:** Angela Taraschi, Costanza Cimini, Alessia Colosimo, Marina Ramal-Sanchez, Fadl Moussa, Samia Mokh, Luca Valbonetti, Giulia Capacchietti, Israiel Tagaram, Nicola Bernabò, Barbara Barboni

**Affiliations:** ^1^ Faculty of Biosciences and Technology for Food, Agriculture and Environment, University of Teramo, Teramo, Italy; ^2^ Istituto Zooprofilattico Sperimentale dell’Abruzzo e del Molise “G. Caporale”, Teramo, Italy; ^3^ Doctoral School of Science and Technology Lebanese University, Beirut, Lebanon; ^4^ National Council for Scientific Research (CNRS), Lebanese Atomic Energy Commission (LAEC), Laboratory for Analysis of Organic Compound (LACO), Beiru, Lebanon; ^5^ Institute of Biochemistry and Cell Biology (CNR-IBBC/EMMA/Infrafrontier/IMPC), National Research Council, Rome, Italy

**Keywords:** diseasome, immune system, oviductal environment, human, biological network, immunological disease, rheumatoid arthritis, asthma

## Abstract

Human hypofertility and infertility are two worldwide conditions experiencing nowadays an alarming increase due to a complex ensemble of events. The immune system has been suggested as one of the responsible for some of the etiopathogenic mechanisms involved in these conditions. To shed some light into the strong correlation between the reproductive and immune system, as can be inferred by the several and valuable manuscripts published to date, here we built a network using a useful bioinformatic tool (DisGeNET), in which the key genes involved in the sperm-oviduct interaction were linked. This constitutes an important event related with Human fertility since this interaction, and specially the spermatozoa, represents a not-self entity immunotolerated by the female. As a result, we discovered that some proteins involved in the sperm-oviduct interaction are implicated in several immune system diseases while, at the same time, some immune system diseases could interfere by using different pathways with the reproduction process. The data presented here could be of great importance to understand the involvement of the immune system in fertility reduction in Humans, setting the basis for potential immune therapeutic tools in the near future.

## 1 Introduction

Fertilization is a cell-cell recognition process that occurs naturally *in vivo* within the oviductal microenvironment of the female body. The successful interaction between the spermatozoa (male gametes) and the oocyte (female gamete) is supported by the presence of oviduct epithelial cells (OECs) and the oviductal fluid, that participates in this complex dialogue either by directly interacting with the gametes (OECs) and secreting (OECs) or carrying (oviductal fluid) different molecules necessary to achieve a successful fertilization.

The process initiates with the arrival of the ejaculated spermatozoa to the cervix, where only the healthiest spermatozoa are selected to advance towards the uterus (or are directly deposed within the uterus, depending on the species), cross the utero-tubal junction and reach the oviduct (reviewed in Suarez, 2016; Gadella, 2017; Li and Winuthayanon, 2017). Here, sperm cells are able to bind to the oviductal epithelium for an indefinite period of time, varying from hours to days (species-specific) and forming the so-called “functional sperm reservoir,” before being released to continue their way towards the oocyte (Suarez and Pacey, 2006; Coy et al., 2012). As a result of this close interaction, it is originated a cross-talk between the OECs and the sperm cells, that is important to ensure the success of early reproductive events (Almiñana, 2015). With regard to the oviductal fluid, it is mainly composed of amino acids, energy metabolites, inorganic salts, glycosaminoglycans and numerous proteins (Ballester et al., 2014; Coy and Yanagimachi, 2015; Canha-Gouveia et al., 2019), that are either passively or actively transported over the epithelial barrier from the circulating blood or the interstitial tissue, or *de novo* secreted by the OECs (Saint-Dizier et al., 2020) and are able to sustain and drive the biochemical machinery of spermatozoa and embryos during their journey.

Thus, on the one hand, the oviduct and its secretions influence the physiology of the gametes (Avilés et al., 2010), while on the other one hand the reproductive cells are able to modulate the oviductal environment by activating a cell-type-specific signalling pathway leading directly to specific alterations in the tubal fluid composition (Georgiou et al., 2007).

Overall, the study of the interaction between the female counterpart with male gametes (firstly) and embryos (secondly) poses a fascinating and challenging questions involving all the hemostatic mechanisms of the body. If the role of neuro-endocrine system is evident, now new emerging evidences are highlighting the involvement of immune system. For instance, the spermatozoa are clearly not-self and the embryos are semi-allogenic, but instead to be attacked by the maternal immune system they are tolerated for days or even months (Zandieh et al., 2015), thus indicating the existence of a gamete recognition system (Georgiou et al., 2007), as will be explained in the discussion section. Moreover, the immune system is involved in the etiopathogenesis of reproductive diseases, as it happens in case of immune/immunological infertility. This condition is diagnosed when spontaneously produced antibodies bind to the antigens occurring on the male gametes, with the production of anti-sperm antibodies (ASA) (Bohring and Krause, 2003; Brazdova et al., 2016).

Ultimately, the involvement of immune system in determining the success of fertility, or its partial or total failure (hypo-fertility or infertility) is still far to be completely deciphered, and the molecules involved in linking reproductive function with immune response are still under investigation.

For this reason, here we carried out an innovative study to explore the possible involvement of genes encoding for proteins that participate to the functional dialogue existing between male gametes and female structures in immune pathologies. In particular, we used an approach based on the application of network theory to the study of biological complexity. By definition a network is a set of nodes (in our care the genes or the diseases) linked by edges (relationship between genes and diseases). The statistical study of network properties will lead to infer biologically relevant information, otherwise hidden by the complexity of the system.

To that, the work was carried as follow: I) retrieving in literature of the proteins involved in the sperm-oviduct interaction; II) creation of the list with the corresponding genes for those proteins; III) linking of the genes to the immune system disease in which it is involved, thus obtaining a bipartite network (a gene-disease network); IV) analysis of the network to infer biologically relevant information; and V) deep analysis of the relevance of this association in animal models of every human immune diseases, which constitutes one of the most valuable experimental approaches used in medical sciences.

The final aim was to suggest new players in the complex relationship between the reproductive function and immune pathology, to shed some light on how fertility could be compromised in immune system dysregulation.

## 2 Materials and Methods

### 2.1 Data Collection

In order to recreate the microenvironment in which fertilization occurs, we collected the scientific literature published between 2005 and December 2020 in peer-reviewed international papers included in Scopus (https://www.scopus.com; accessed on 20/09/2021). In parallel, and as a quality control, two qualified researchers used the same key-words (“protein” AND “oviductal secretion” or “oviduct”), to carry out an independent search on the published manuscripts including information about the proteins found in the human oviduct. Then, the databases were compared, and a third qualified researcher verified the correctness of the record inserted, resolving eventual conflicts.

Data from each independent search was extracted to Excel spreadsheets (Microsoft Corporation, Albuquerque, USA), filling in and the following fields:

• *Species:* human;
• *Protein:* protein found in oviductal environment;
• *Gene:* protein-related gene;
• *Biological function:* physiological and/or pathological role of the protein;
• *Role in fertilization:* physiological and/or pathological role of the protein related to fertility;
• *OF/OEC/oviductal tissue:* protein identified within the oviductal fluid, on/in the oviductal epithelial cells or oviductal tissue;
• *References:* article reporting the above-mentioned data;
• *Phenotype ko mice:* existence of KO mouse and its relative phenotype;
• *Notes:* any further information useful for the study.

These data can be found in Supplementary Material S1.

### 2.2 Diseasome Creation and Visualization

Bioinformatics analysis was performed using Reactome, DisGeNET Cytoscape App, and Cytoscape 3.7.2.

First, we uploaded the gene list to Reactome (http://www.reactome.org/; accessed on 11/10/2021), a free, open-source, curated and peer-reviewed pathway database useful to visualize and analyse the biochemical pathways in which the genes are involved.

DisGeNET is a Cytoscape plugin designed to analyze human gene–disease association (GDA) networks, the diseasome. GDA is represented as a bipartite graph in which a set of nodes consists of diseases and the other one of disease-associated genes (Bauer-Mehren et al., 2010; Pavlopoulos et al., 2018). A disease and a gene are connected by a link only if the gene is implicated in the particular disease (Pavlopoulos et al., 2018). DisGeNET integrates information on human diseases and their genes from expert curated repositories, GWAS catalogues, animal models and the scientific literature discovered by text-mining approaches (Pinero et al., 20152015; Piñero et al., 2017; Piñero et al., 2020). Data are organized according to the type of source databases:

• CURATED: gene-disease association provided by expert curated resources, such as UniProt, ClinGen, Orphanet and CTD (human data), among others (Piñero et al., 2020);
• ANIMAL MODELS: gene-disease association provided by resources containing information about animal models (currently rat and mouse) of disease (RGD, MGD, and CTD) (Piñero et al., 2020);
• INFERRED: gene-disease association from the Human Phenotype Ontology and from VDAs reported by Clinvar, the GWAS catalogue and GWAS db (Piñero et al., 2020);
• ALL: gene-disease association from the previous sources and from LHGDN and BeFree (Piñero et al., 2020).

In addition, DisGeNET is able to classify the diseases according to the MeSH hierarchy and the genes according to the PANTHER Protein Class Ontology and Reactome top-level pathways (Pinero et al., 20152015). The gene-diseases associations are classified according to the DisGeNET association type ontology, that describes the different types of association between a gene and a disease, integrating information from the different databases (Bauer-Mehren et al., 2011). The GDA ontology is available at https://www.disgenet.org/dbinfo (accessed on 20/05/2021).

Using the DiGeNET Cytoscape App, we built two different networks for each gene in “Gene Disease Networks” tab, selecting “curated” or “animal models” as sources and “Immune System Diseases” as disease class. After merging the obtained networks on Cytoscape, we built two final diseasomes: the first curated (CURDi) and the second referred to animal models (AMDi). Both were then analysed using the plugin Network Analyzer.

### 2.3 Network Creation, Visualization, and Analysis

As previously stablished, the diseasome network was realized and analyzed using Cytoscape 3.7.2 and the specific plug-in Network Analyzer.

## 3 Results and Discussion

### 3.1 Proteins Involved in the Sperm-Oviduct Interaction

**FIGURE 1:**
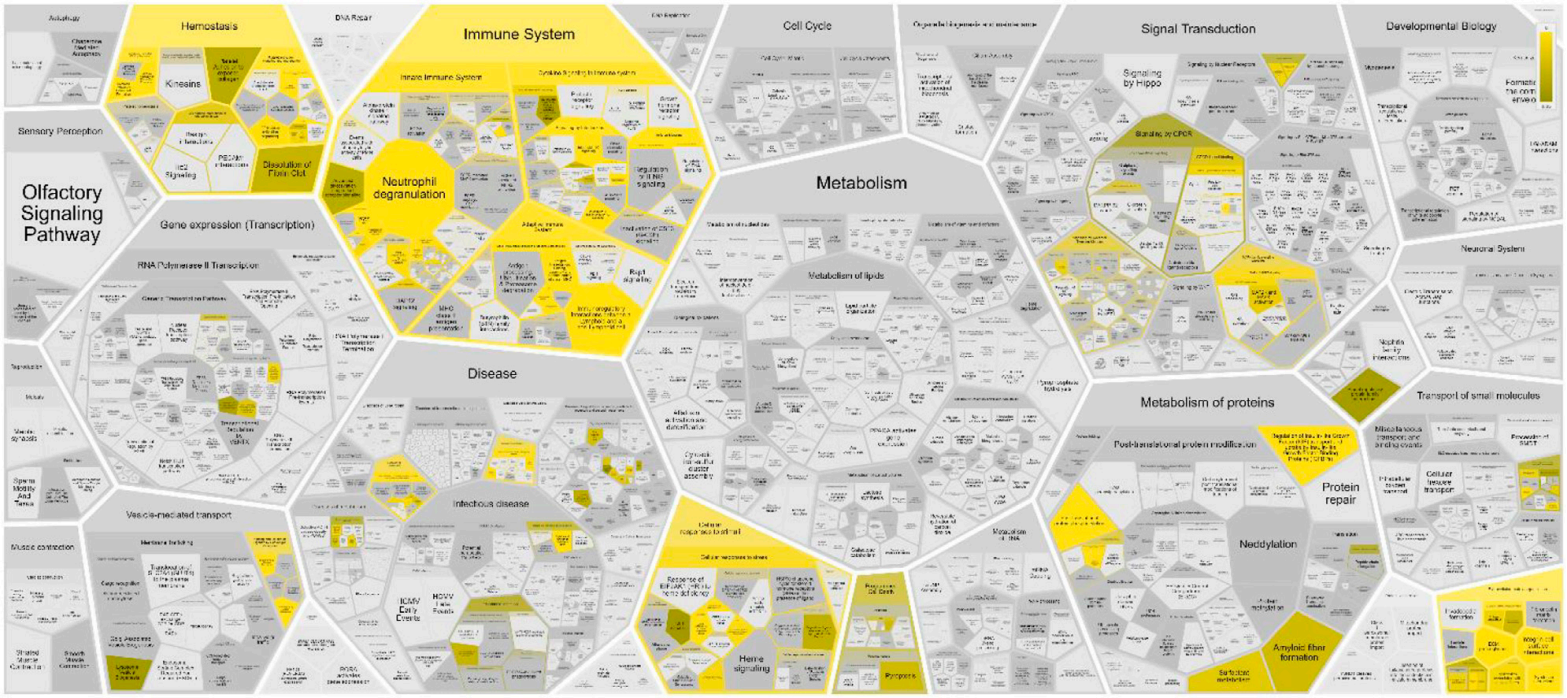
Voronoi pathway visualization (Reacfoam) for the identified proteins in human oviduct. The color code denotes over-representation of that pathway in our input dataset. Light grey signifies pathways which are not significantly over-represented.

The sperm-oviduct interaction and fertilization process can be considered as complex systems constituted by networks of heterogeneous elements interacting among them in a non-linearly way, giving rise to an emergent behavior. Thus, their properties cannot be explored or predicted simply by analysing their individual components, rather by putting their individual pieces togheter and building a network model. To this aim, a total of 145 proteins were identified through the literature search as proteins expressed within the oviduct and involved in the sperm-oviduct interaction in humans (see Supplementary Material S1, second sheet for the list of proteins and their corresponding genes, LOPaG). Here, we have used Reactome to investigate the pathways in which the identified proteins are involved. The analysis showed the 25 most relevant immunology pathways (see Figure 1; Supplementary Material S2), stressing the strong correlation between reproduction and immune system.

Then, by using the DisGeNET Cytoscape App and the genes list, we realized a bipartite network, i.e., a graph constituted by two families of nodes (genes and immune diseases) connected by edges and that represent the gene-disease association.

**FIGURE 2:**
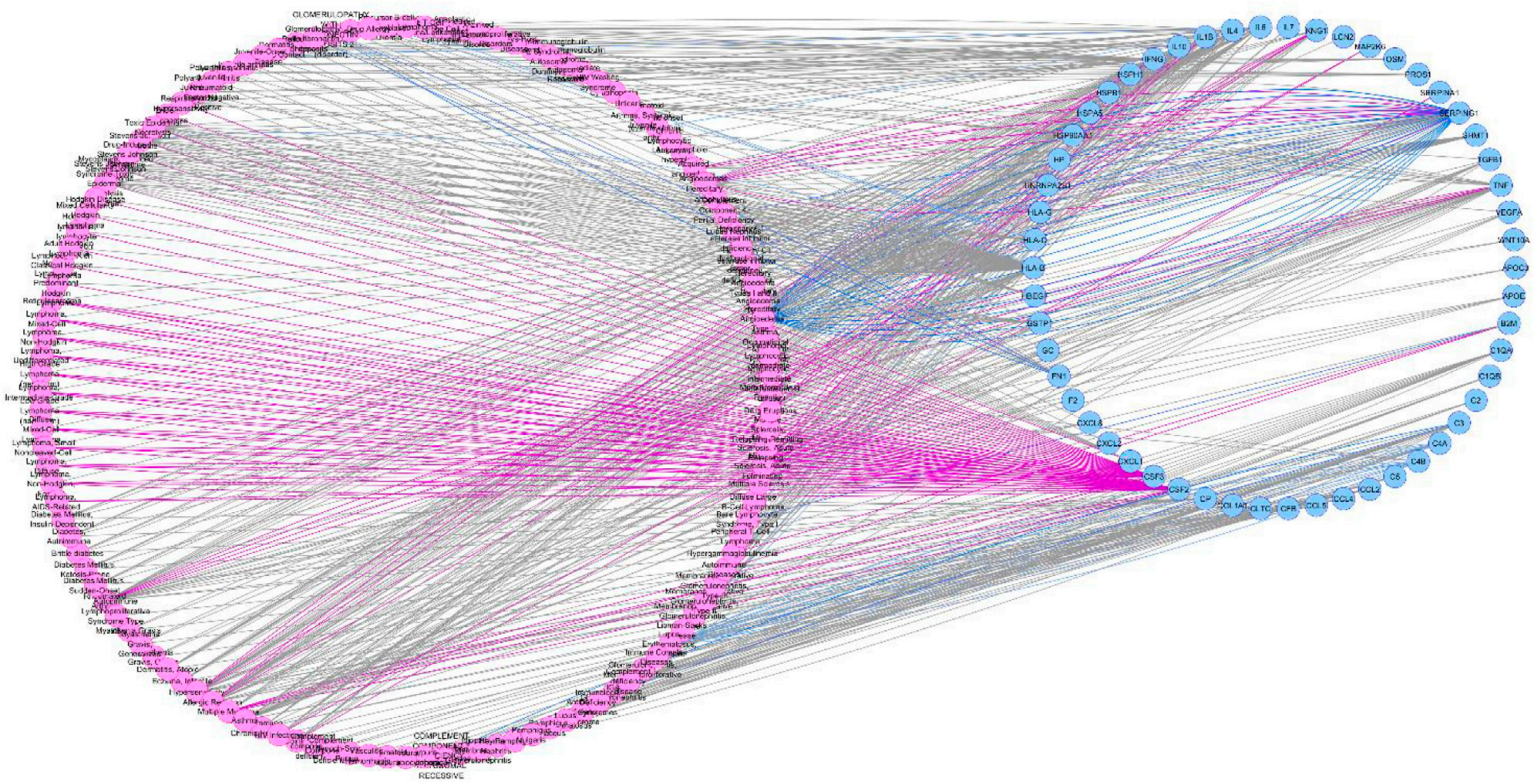
Curated diseasome network (CURDi). CURDi forms the two node sets of bipartite networks with two types of nodes: diseases (pink circle) and gene (blue circle). Disease node and gene node are connected if the gene is implicated in the disorder.

**FIGURE 3:**
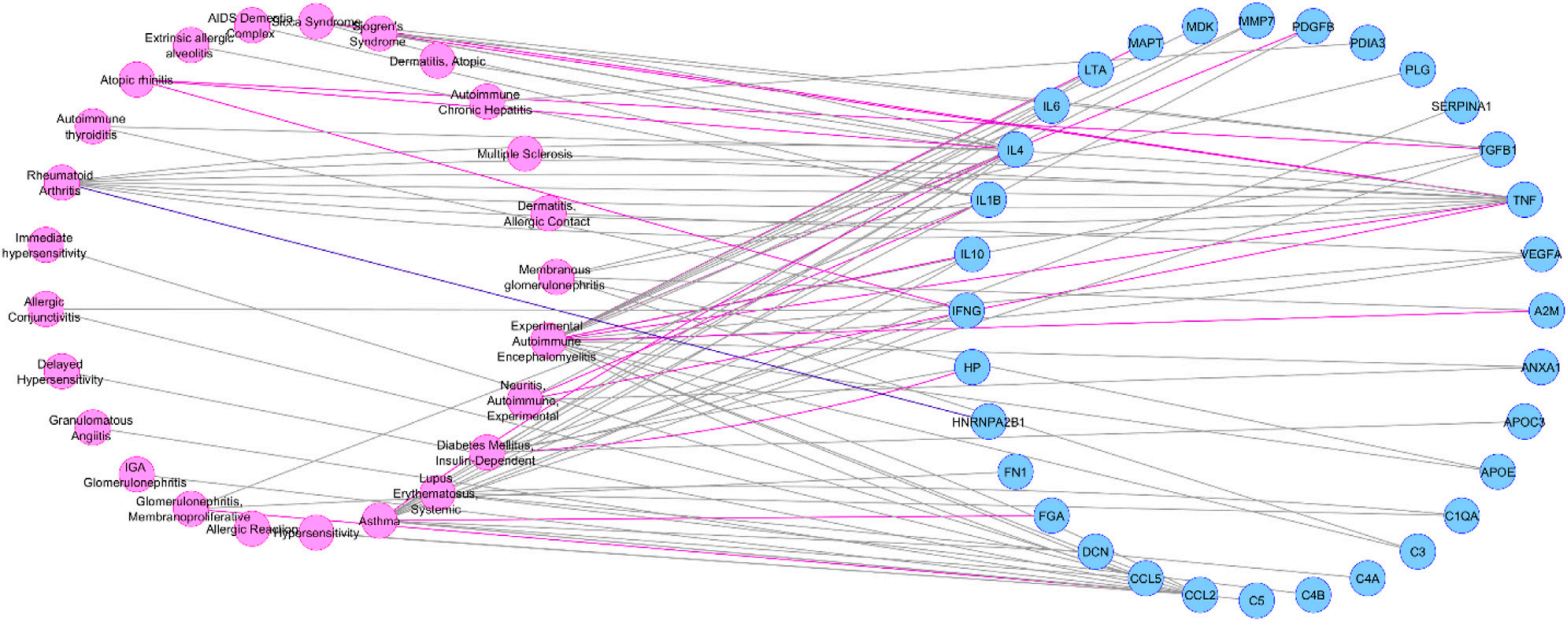
Animal model diseasome network (AMDi) ***.*** AMDi form the two node sets of bipartite networks with two types of nodes: diseases (pink circle) and gene (blue circle). Disease node and gene node are connected if the gene is implicated in the disorder.

Depending on the data source (Curated or Animal Models Archives) we obtained two different diseasome networks: curated diseasome network (CURDi, see Figure 2) and animal model diseasome network (AMDi, see Figure 3).

### 3.2 CURDi Network and the Most Linked Genes

In CURDi network 54 of the 145 genes present in LOPaG were correlated with 124 immune system diseases.

**FIGURE 4:**
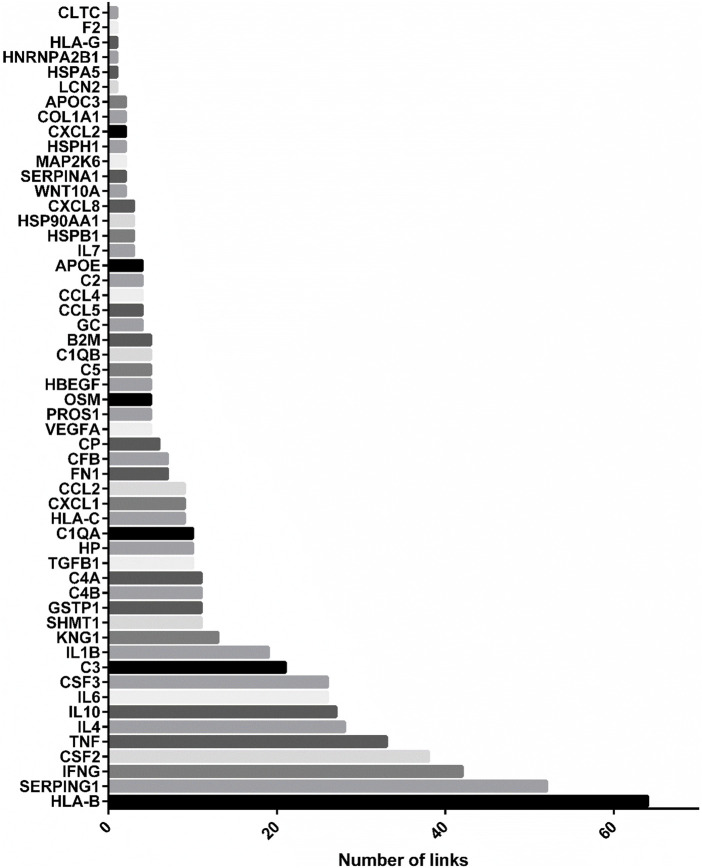
Most linked genes in CURDi network. The histograms show the most linked genes to immune system diseases in CURDi: HLA-B, SERPING1 and IFNG.

As showed in Figure 4, the most linked genes in CURDi were *HLA-B, SERPING1* and *IFNG* (64, 52, and 42 links each one, respectively) (see Supplementary Material S3, sheet 1 for the complete list). These genes are well-studied for their key role in the immune response since their alteration may be responsible for several immune system diseases. Interestingly, there is growing evidence on the roles played by proteins encoded by the *HLA-B, SERPING1* and *IFNG* genes in several steps of the reproduction process.

#### 3.2.1 HLA-B Gene

Among the 54 genes correlated with immune system diseases within the CURDi network, HLA-B stands out as the most linked one. This gene encodes for the human leukocyte antigen type B (HLA-B), one of the more than 200 genes belonging to the major histocompatibility complex (MHC) in humans. Located on chromosome 6p21.3, it comprises specific HLA class I (HLA-A and -B) and class II (HLA-DRB1, -DQA1, -DQB1, -DPA1 and -DPB1) genes that encode for cell-surface glycoproteins, whose main action is the induction and regulation of immune response (Leone et al., 2013; Wieczorek et al., 2017; Jongsma et al., 2019).

The genes of the MHC are the most polymorphic of the human genome with a total of 13,023 HLA alleles (HLA class I: 9749; HLA class II: 3274) (Robinson et al., 2015). Interestingly, distinct HLA alleles have been associated with several human pathological conditions (Tersigni et al., 2020), while HLA proteins also own an important role in non-pathological conditions, such as lifespan and social behavior (Mosaad, 2015).

Regarding more Specifically, different alleles of the HLA-B gene have been associated with autoimmune diseases (such as HLA-B27 and its relationship with psoriatic arthritis and ankylosing spondylitis), inflammatory diseases (such as HLA-B*35 and systemic sclerosis, and HLA-B*52 and Takayasu arteritis), viral infections (such as HLA-B*35 phenotype and progression of Acquired Immune Deficiency Syndrome-AIDS) and tumor risks (such as HLA-B*52:01 and cervical cancer). In addition, it has been demonstrated an association of HLA-B alleles and severe drug hypersensitivity syndromes (such as HLA-B*57:01 and hypersensitivity to abacavir, and HLA-B*15:02 and use of carbamazepine) (Profaizer and Eckels, 2012).

In the reproductive field, the HLA antigens have been demonstrated to be crucial for the embryo-maternal tolerance and the achievement of a successful pregnancy (Chattopadhyay et al., 2014; Tersigni et al., 2020). For instance, some molecules as the high polymorphic HLA-C participate in the innate immune system by serving as a ligand for the inhibitory killer cell immunoglobulin-like receptors (KIRs) present on natural killer (NK) cells (Leone et al., 2013; Wilczyńska et al., 2020). HLA-C (along with the HLA-E, G and F ones) from both maternal and paternal origin is highly expressed by the extravillous trophoblasts invading the uterine tissues. While the paternal HLA-C protein represents a main target for maternal NK and T cells, an increased expression of foreign HLA-C (as in the case of oocyte donation) can be correlated with an incorrect placentation and further linked pathologies, thus requiring a tight regulation in the dual function of the protein (Papúchová et al., 2019). Despite the absence of evidence regarding the direct involvement between HLA-B and the immune response in the embryo, it might be possible to hypothesize that the close link between HLA-B and the encoding area of HLA-C could exert an indirect effect in the interaction between the NK cells from the uterus and the trophoblast HLA-C (Nielsen et al., 2017).

In addition, discordant results have been reported so far on the role of HLA polymorphisms on the susceptibility to pre-eclampsia (PE) (Emmery et al., 2016). This complex disease, exclusive to human pregnancy, shows clinical features as a new onset of hypertension and proteinuria after 20 weeks of gestation and is characterized by a systemic disproportionated inflammatory response, representing the main cause of maternal and perinatal morbidity and mortality with a prevalence of 3–8% in the total number of pregnancies worldwide and an increasing incidence. The four main potential causes underlying the pathophysiology of pre-eclampsia include: an immunological maladaptive tolerance between maternal, paternal, and fetal tissues; placental implantation with abnormal trophoblastic invasion; oxidative stress causing endothelial cell dysfunction; and genetic and epigenetic predisposing alterations (Agius et al., 2018). Regarding the immunological maladaption occurring between mothers and fetuses, few studies have focused on the role of HLA alleles in inducing pre-eclampsia. Wiktor and collaborators reported a significant increase of HLA-B13 allele frequency in patients with pre-eclampsia and of HLA-B22 allele in their male partners (Wiktor and Kozioł, 1998). A subsequent study of Zhang Z et al. in 119 Chinese pre-eclamptic patients showed a higher frequency of some HLA alleles shared by mothers and fetuses (HLA-A11, HLA-B13, HLA-B15, HLA-B22), and a lower frequency of a different protective allele (HLA-B14) (Zhang et al., 2009). On the contrary, a study carried out in 201 Danish couples of mothers and children reported no specific association with HLA-A, -B, and -DR alleles, denying the role of HLA antigens as risk factors for pre-eclampsia (Biggar et al., 2010). An association of HLA-G polymorphic alleles with pre-eclampsia has also been reported in several studies (Moreau et al., 2008; Tan et al., 2008; Persson et al., 2017).

Recently, a more comprehensive report of genome-wide association (GWAS), transcriptomics, proteomics and metabolomics studies identified inhibin as a potential preeclamptic biomarker (Benny et al., 2020).

Despite few studies have focused on the role of HLA alleles in inducing pre-eclampsia, further functional studies are necessary to clarify an effective role of the classical HLA genes in its etiopathogenesis.

#### 3.2.2 SERPING1 Gene

The second most linked gene, SERPING1, encodes for the plasma protease serine inhibitor (C1-INH), also known as SERPING1 or C1-inhibitor (Madsen et al., 2014). C1-INH regulates the activation of the classical and lectin complement pathways, coagulation and fibrinolysis cascades (López-Lera et al., 2014). Mutations in the SERPING1 gene are responsible for the largest cases of hereditary angioedema (HAE) (OMIM#106100), a rare autosomal dominant disorder that causes recurrent attacks of cutaneous angioedema, severe abdominal pain, and airway compromise (Santacroce et al., 2021). The disease course during pregnancy is unpredictable, with one study showing that seven Australian patients with HAE had reduced or absent attacks in the last two trimesters of pregnancy, while in the post-partum period they suffered from increased frequency and more severe attacks (Chinniah and Katelaris, 2009). However, fertility seems not to be impaired by HAE itself or by HAE medications (Yakaboski et al., 2020).

A network study by Sabetian and coll. (2014) built a sperm and oocyte protein interaction network and revealed new protein interactions. For example, the authors indicated that SERPINE1, also known as PAI-1 (plasminogen activator inhibitor), is located on the surface, in the tail and in the acrosome of mature spermatozoa, participating in the sperm-egg interaction by interacting with C1-INH of the oocyte (Sabetian et al., 2014). Thus, our results suggest that new studies could be useful to better clarify the interactions among the SERPING1 gene, immune diseases and fertility.

#### 3.2.3 INFG Gene

The IFNG gene codifies for an extracellular proinflammatory cytokine (interferon γ, IFN-γ) that constitutes the main effector of cell-mediated immunity. Its main function is to recognize and eliminate pathogens by enhancing the antigen recognition through the antigen presenting cells and T cells, and is secreted by CD4^+^, NK and NKT cells. It is able to intervene as the early host defense and autocrine regulation but also during the adaptative immune response (reviewed in (Schroder et al., 2004; Bhat et al., 2018; Kak et al., 2018)).

In reproduction, IFN-γ shows an important role on embryo implantation and pregnancy progression (Robertson et al., 2018). For instance, increased levels of IFN-γ have been associated with a reduced fertility (Carrasquel et al., 2014), as evidenced by the results of Carrasquel and coll. (2014). In that *in vitro* study, high concentrations of IFN-γ affected the intracellular calcium concentration, altering the sperm membrane permeability and thus impairing the sperm fertilizing ability (Carrasquel et al., 2014). Moreover, it has been demonstrated that an excess of the protein can also promote the generation of cytotoxic or CD8^+^ cells during the embryo implantation that later drives to fetal loss (Robertson et al., 2018), thus supporting its fundamental involvement as a regulator of the maternal-fetal immune relationship.

Being secreted in the uterus during early pregnancy, IFN-γ plays a critical role in gestation, including remodeling of endometrial vasculature, angiogenesis at implantation sites, and maintenance of the decidual (maternal) component of the placenta. Alteration of INF-γ levels in the plasma of pregnant women may contribute to severe gestational pathologies, such as autoimmune disease, preterm labor, and preeclampsia (Sargent et al., 2006; Murphy et al., 2009; Yang et al., 2014).

One plausible mechanism could be the inability of the mother to switch from T helper cell type 1 (Th1) to Th2 cytokine profiles at the fetal-maternal interface, due to an altered expression of INF-γ and its receptors (IFN-γ R1 and IFN-γ R2) (Sargent et al., 2006).

#### 3.2.4 Other Genes

In the list of most connected genes, CSF2 showed 38 links. This gene encodes for the granulocyte-macrophage colony-stimulating factor (GM-CSF), responsible for the growth and differentiation of hematopoietic precursor cells in granulocytes, macrophages, eosinophils and erythrocytes, among others. Interestingly, an important role has also been given to this protein during the fertilization process. Specifically, GM-CSF was found to mediate the maternal effects on embryonic development during preimplantation, probably by inducing the expression of IFN-γ (Loureiro et al., 2009). The presence of GM-CSF receptors has been also described in the midpiece and principal segment of the tail of mature spermatozoa in human and bovine species, while it was also demonstrated that GM-CSF was able to improve sperm motility when added to bovine sperm samples (Vilanova et al., 2003). In Csf2 null mutant mice, a deficiency in GM-CSF protein levels resulted in altered differentiation and maturation of junctional-zone trophoblast lineages, glycogen cells, and giant cells, thus suggesting the role of the Csf2 gene as a regulator of trophoblast differentiation and placental development (Sferruzzi-Perri et al., 2009).

Among the other most connected genes in the CURDi network stand out several genes codifying for cytokines, such as the tumor necrosis factor alpha (TNF-α), interleukins 4, 6 and 10 (IL-4, IL-6 and IL-10, respectively), and granulocyte colony-stimulating factor (G-CSF). TNF-α is a cytokine codified by the TNF gene and with a wide variety of functions. It is naturally produced by activated macrophages and monocytes, and its increased levels have been associated with infertility in humans (Eggert-Kruse et al., 2007; Yildizfer et al., 2015; Pinto-Bravo et al., 2017). Although few studies evaluated the role of TNF-α in the oviduct, evidence support that TNF-α may modulate the oviduct contraction necessary for transporting the gametes and embryo into the site of fertilization and the uterus, respectively (Wijayagunawardane et al., 2003; Parada-Bustamante et al., 2016). In addition, increased levels of TNF-α was detected in the tubal fluid of patients with hydrosalpinx and salpingitis due to chlamydial or gonococcal infection (Nasu et al., 2007). In these pathological conditions, TNF-α may induce the vascular endothelial growth factor (VEGF) production, which may further enhance the oviductal secretion by regulating vascular permeability (Nasu et al., 2007).

Interleukin-4 and -10 are pleiotropic anti-inflammatory cytokines that function mainly by suppressing the pro-inflammatory milieu (Chatterjee et al., 2014). For this reason, they play crucial roles in the success of pregnancy: progesterone induces the IL-4 and IL-10 production, which acts to inhibit Th1 responses during pregnancy, creating a tolerogenic environment in women (Chatterjee et al., 2014; Shahbazi et al., 2019). Indeed, while the trophoblastic cell implantation into endometrial cells is associated with an active Th1 pro-inflammatory response, the pregnancy maintenance is marked by an anti-inflammatory response, promoting fetal allograft tolerance and ensuring fetal development (Granot et al., 2012; Chatterjee et al., 2014).

Interleukin-6 is a pleiotropic cytokine involved in both acute and chronic inflammatory processes (Papathanasiou et al., 2008; Balasubramaniam et al., 2012). Papathanasiou and coll. (2008) showed that IL-6, in addition to act as an inflammatory marker, is capable *in vitro* to significantly reduce the ciliary beat function (CBF) causing a severe tubal damage, whereas the addition of anti-IL-6 restores the activity of CBF (Papathanasiou et al., 2008). IL-6 may also play a role in the pathophysiology of tubal ectopic gestation. Indeed, it was demonstrated that the expression of IL-6 is significantly increased near the implantation site in tubes with ectopic gestation, as compared with normal gestations (Balasubramaniam et al., 2012). On the other hand, IL-6 has been shown to affect sperm motility and to induce protein tyrosine phosphorylation in human spermatozoa (Laflamme et al., 2005).

Granulocyte-colony stimulating factor (G-CSF) is a pleiotropic cytokine belonging to the hematopoietic growth factor family that codifies by the CSF3 gene. Recent studies has revealed granulocyte colony-stimulating factor (G-CSF) as a predictive biomarker of oocyte and embryo developmental competence in humans (Naghshineh et al., 2018; Cai et al., 2020), promoting endometrial thickening and improving the pathophysiology of endometriosis, which all fundamentally lead to preventing from the pregnancy loss (Cai et al., 2020).

### 3.3 CURDi Network and the Most Linked Immune Diseases

Analyzing the link of the selected gene set with diseases involving other organs and systems (different from the Immune System), we found that the largest number of pathologies were related to the following groups: “Hemic and Lymphatic diseases” (Chinniah and Katelaris, 2009), “Skin and Connective Tissues diseases” (Emmery et al., 2016) and “Neoplasms” (Nielsen et al., 2017) (Table 1; Supplementary Material S3, second sheet for the complete dataset).

**TABLE 1:** Group of diseases and number of diseases included within each group.

Class	Disease class	Number of diseases
*C15*	Hemic and Lymphatic Diseases	45
*C17*	Skin and Connective Tissue Diseases	33
*C04*	Neoplasms	32
*C16*	Congenital, Hereditary and Neonatal Diseases and Abnormalities	14
*C14*	Cardiovascular Diseases	13
*C12*	Male Urogenital Diseases	11
*C13*	Female Urogenital Diseases and Pregnancy Complications	11
*C05*	Musculoskeletal Diseases	8
*C25*	Chemically-Induced Disorders	8
*C10*	Nervous System Diseases	7
*C18*	Nutritional and Metabolic Diseases	7
*C23*	Pathological Conditions Signs and Symptoms	7
*C19*	Endocrine System Diseases	6
*C01*	Infections	5
*C07*	Stomatognathic Diseases	5
*C08*	Respiratory Tract Diseases	3
*C06*	Digestive System Diseases	1
*C11*	Eye Diseases	1
*C24*	Occupational Diseases	1

**FIGURE 5:**
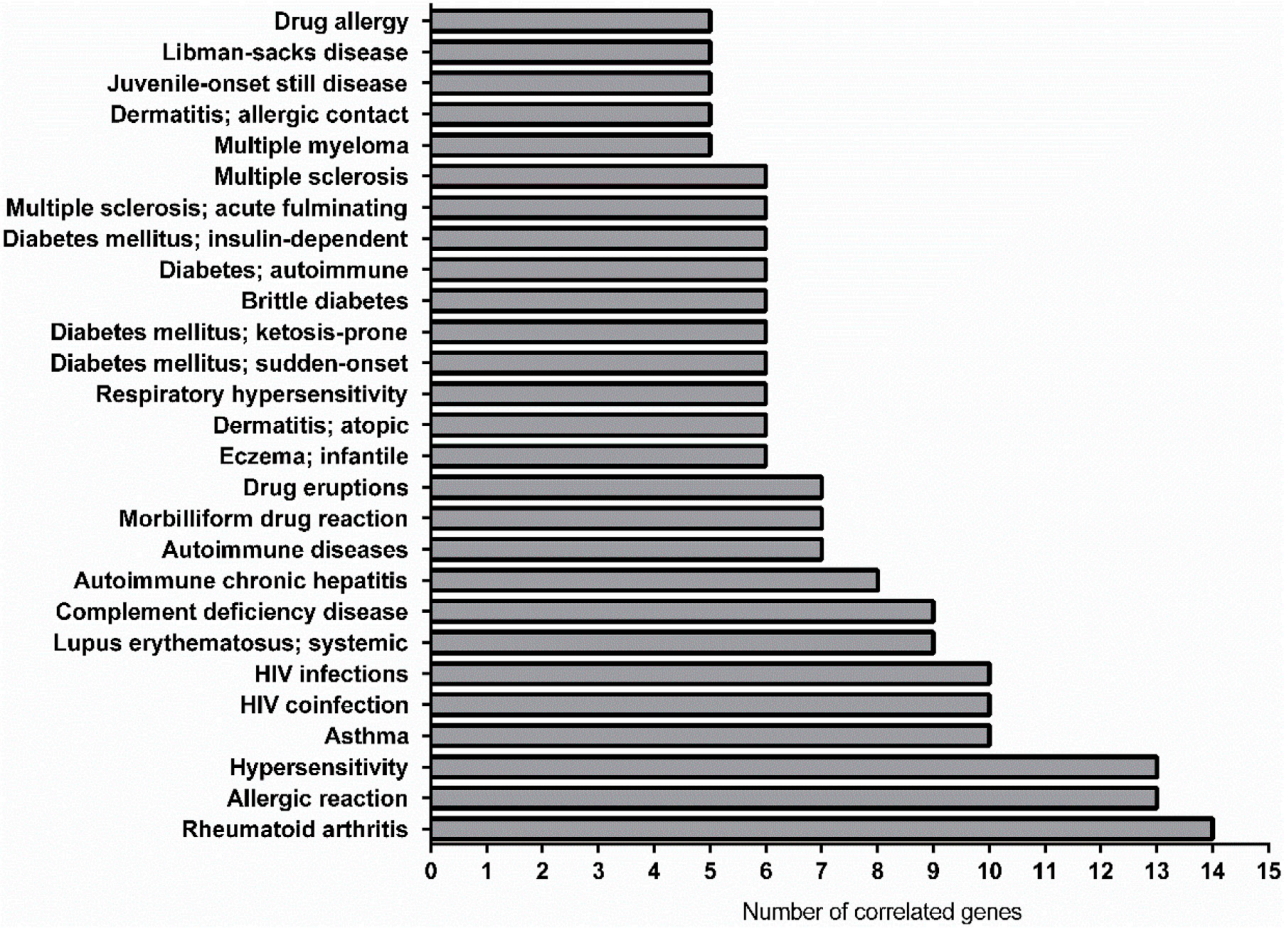
Graphical representation of the most linked immune system diseases with the gene list in CURDi. The highest number of correlated genes are found in rheumatoid arthritis (14 linked genes), allergic reaction and hypersensitivity (13 linked genes) and asthma (10 linked genes).

As showed in Figure 5 the most linked diseases to the list of genes from CURDi were rheumatoid arthritis (14 linked genes), allergic reaction and hypersensitivity (13 linked genes) and asthma (10 linked genes) (for the complete list of diseases and related information see Supplementary Material S3, sheet 3).

#### 3.3.1 Rheumatoid Arthritis

Among the three most linked conditions, only rheumatoid arthritis (RA) has been related to fertility (Fattah et al., 2020) so far, maybe because the other two (i.e., allergic reaction and hypersensibility) show very high variability and multiple interconnected components. A recent review by Fattah and coll. (2020) provided several proofs regarding the relationship between women with RA and fertility, which seems declined and dependent on inflammatory milieu, mother age, hampered sexual activity and negative effects of non-steroidal anti-inflammatory drugs on ovarian function (Fattah et al., 2020). Indeed, it has been found that women with RA deliver fewer children when compared to healthy women (Fattah et al., 2020). The decreased fertility rate in women suffering from RA might be due to a reduced sexual activity (because of pain, fatigue, mental distress, functional limitations), treatment with antirheumatic medications hampering ovulation, as well as, to advanced maternal age, patients’ choice, or a combination of all of these factors (Fattah et al., 2020). The results showed here demonstrate that at least 13 genes (CXCL8; CSF2; IL6; LCN2; TNF; VEGFA; IFNG; IL1B; IL10; CP; CXCL2; GC; F1) could be involved in this relationship.

#### 3.3.2 Asthma

From the CURDi analysis, asthma showed 10 linked genes. The link between asthma and infertility was studied in a nationwide register-based twin study, in which a cohort of 15,250 twins living in Denmark participated in a questionnaire study including questions about the presence of asthma and fertility (Gade et al., 2014). Differences in time to pregnancy and pregnancy outcome were analysed in subjects affected with asthma and allergy and in healthy individuals, using multiple regression analysis. Results showed an association between asthma and an increased time to pregnancy, with a percentage of asthmatics with a time to pregnancy >1 year of 27% versus the 21.6% for the non-asthmatic individuals. Interestingly, the association remained significant after adjustment for age, age at menarche, body mass index and socioeconomic status and was more pronounced in those >30 years of age. In addition, untreated asthmatics had a significant increased risk of prolonged time to pregnancy compared to control individuals, while asthmatics receiving any kind of treatment for asthma tended to have a shorter time to pregnancy than untreated asthmatics (Gade et al., 2014). Thus, the authors concluded that asthma seems to be correlated with an alteration in fertility parameters, and that the negative effect of asthma on fertility increases with age and disease severity.

### 3.4 AMDi Network and the Most Linked Genes

Since the study of human diseases takes a huge advantage by the use of animal models as valuable resource for the investigation of pathogenesis, diagnostics, and therapeutics of human diseases, we realized the network representing the connections between the selected gene set and the immune diseases in animal models (AMDi). The most linked genes were IL4, TNF and CCL2, (12, 12 and 10 links, respectively) (see Supplementary Material S4, sheet 1 for the complete list).

The roles of IL4 and TNF, have been discussed before. The CCL2 gene codifies for the small chemokine CCL2, also referred to as monocyte chemotactic protein 1 (MCP1), which is secreted by endothelial, epithelial and stromal cells, monocytes and lymphocytes (Hess et al., 2013). It influences the innate immunity through its effects on monocytes, as well as the adaptive immunity through the control of T helper cell polarization (Hess et al., 2013). It was proposed that chemokines expressed by the oviductal epithelial cells contribute to normal physiological homoeostasis and protection from pathogens by activating the immune cells (Fahey et al., 2005). In addition to this protective function, chemokines, including CCL2, may protect these cells from malignant transformation, again suggesting that CCL2 may be involved in early tumour development (Wojnarowicz et al., 2012). It was also shown that a marked down-regulation of CCL2 may contribute to allogenic tolerance of the preimplantation embryo as it crosses the Fallopian tube (Hess et al., 2013).

Interestingly, an association between two CCL2 polymorphisms (rs1024611 and rs4586) and the development of gestational diabetes mellitus (GDM), the most common medical complication of human pregnancy, was demonstrated in 411 pregnant women (Teler et al., 2017). To this regard, a more recent study confirmed that blocking the CCL2/CCR2 pathway in a mouse GDM model, the inflammatory cytokines may be reduced, mitigating GDM symptoms and improving the reproductive outcomes in mice (Qi et al., 2021).

### 3.5 AMDi Network and the Most Linked Diseases

**FIGURE 6:**
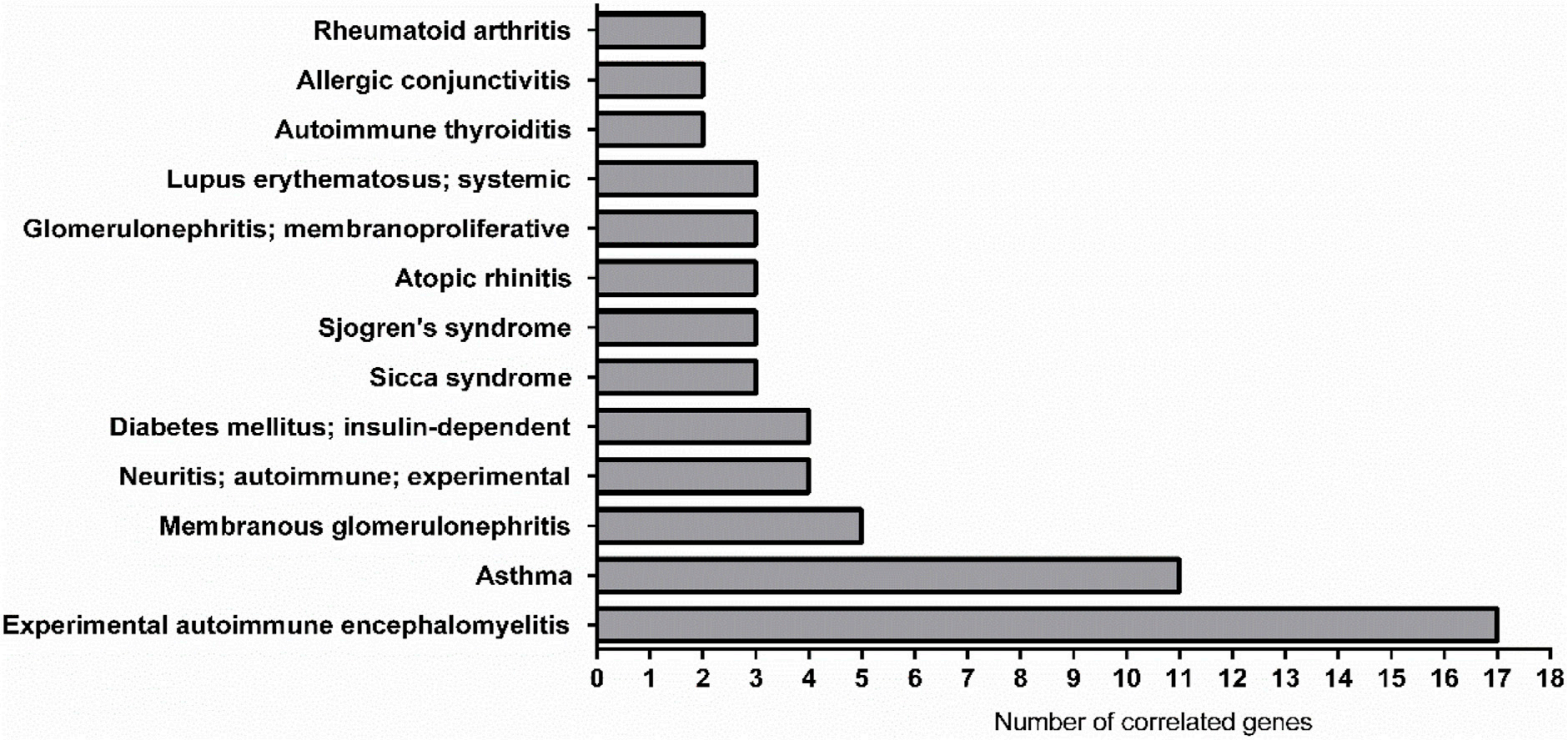
Graphical representation of the most linked immune system diseases with the genes list in AMDi. The most linked diseases were experimental autoimmune encephalomyelitis (EAE, linked 17 genes) and asthma (linked 11 genes).

The AMDi network also provided very intriguing and useful information. For instance, the two pathologies related with the highest number of correlated genes are the Experimental Autoimmune Encephalomyelitis (EAE, 17 genes) and Asthma (11 genes), this last being already discussed above (for the complete list of diseases and related information, see Figure 6; Supplementary Material S4, second sheet).

#### 3.5.1 Experimental Autoimmune Encephalomyelitis

EAE is an autoimmune encephalomyelitis commonly used as an experimental model for the human inflammatory demyelinating disease, multiple sclerosis (MS). It constitutes a complex condition in which the interaction between a variety of immunopathological and neuropathological mechanisms leads to the key pathological features of MS: inflammation, demyelination, axonal loss and gliosis (Constantinescu et al., 2011).

The exploration of the link between MS and infertility is very complex for several reasons. As discussed by Cavalla and coll. (2006), the frequency of childlessness in the female MS patients seems to be higher than in the general population (Cavalla et al., 2006). Rather than lowered fertility, this could reflect other issues related to this pathology, such as the fact that patients may choose to avoid or postpone pregnancy, mainly because of concern about taking care of the baby or about the risk of transmitting a genetic susceptibility to MS to their children (Cavalla et al., 2006). A recent study has shown that women affected with MS had lower live birth rates (LBR) compared to unaffected women (irrespective of their infertility diagnosis or treatment) (Houtchens et al., 2020). This statistically significant difference in LBRs was more evident in women in early (Bauer-Mehren et al., 2011; Profaizer and Eckels, 2012; Leone et al., 2013; Chattopadhyay et al., 2014; Mosaad, 2015; Pinero et al., 20152015; Robinson et al., 2015; Piñero et al., 2017; Wieczorek et al., 2017; Jongsma et al., 2019; Piñero et al., 2020; Tersigni et al., 2020; Wilczyńska et al., 2020) and middle (Emmery et al., 2016; Nielsen et al., 2017; Agius et al., 2018; Papúchová et al., 2019) childbearing years. The difference between women with and without MS disappeared after receiving infertility treatments, thus highlighting the importance of information regarding the efficacy of infertility treatments in women with autoimmune diseases (Houtchens et al., 2020).

Despite the fact that MS is three times more common in women than in men and that endocrine alteration commonly found in MS patients and immunosuppressive therapies could interfere with fertility, Glazer and co-workers evaluated the association of MS and male infertility in a register-based cohort study in Denmark between 1994 and 2015 (Glazer et al., 2017). A comparison was made between a group of 24,011 men diagnosed with male factor infertility and a control group of 27,052 normal males. Infertile men showed a higher risk of prevalent and incident MS when compared to the reference group, thus suggesting, for the first time, an association between male infertility and MS (Glazer et al., 2017).

Here we provided the evidence that in both EAE and asthma a common genetic background could explain, at least in part, the finding that a systemic inflammation can also involve the reproductive system.

From the results obtained in this study, we highlighted as immune system and reproductive function are closely linked. Indeed, as it was shown, some proteins involved in sperm-oviduct interaction could be involved in several immune system diseases, while, at the same time, some immune system diseases could interfere with the reproduction process, although their causal relationship is still unclear.

However, to better understand the cross-talk between the immune and the reproductive systems are needed further investigations, such as wider epidemiological studies and experimental research with the use of animal models.

In conclusion, our innovative approach fits well in the field of “reproductive immunology” that represents an active area of research aimed at understanding how the immune system contributes to human reproduction. In a clinical research scenario this comprehension might be fundamental in reducing implantation failure and recurrent miscarriage in assisted reproductive technologies (ARTs).

## Data Availability

The original contributions presented in the study are included in the article/Supplementary Material, further inquiries can be directed to the corresponding author.
