# Supplementary figures and images for: Human Immune System Diseasome Networks and Female Oviductal Microenvironment: New Horizons to be Discovered
Source: Front Genet. 2022 Jan 27;12:795123. doi: 10.3389/fgene.2021.795123 (PMC8829125; doi:10.3389/fgene.2021.795123)

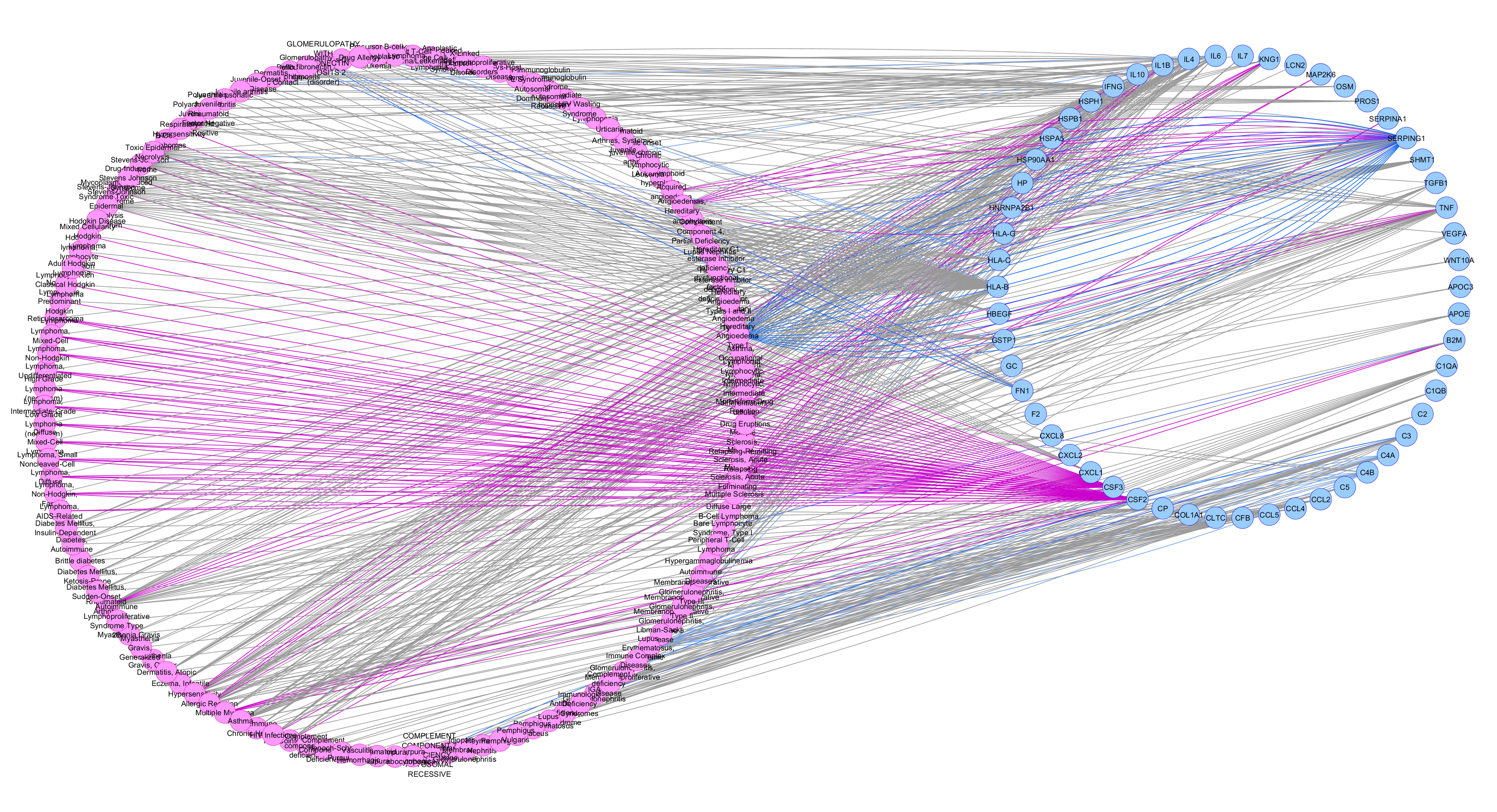

Supplement: Supplementary file 2 [file Image3.JPEG]

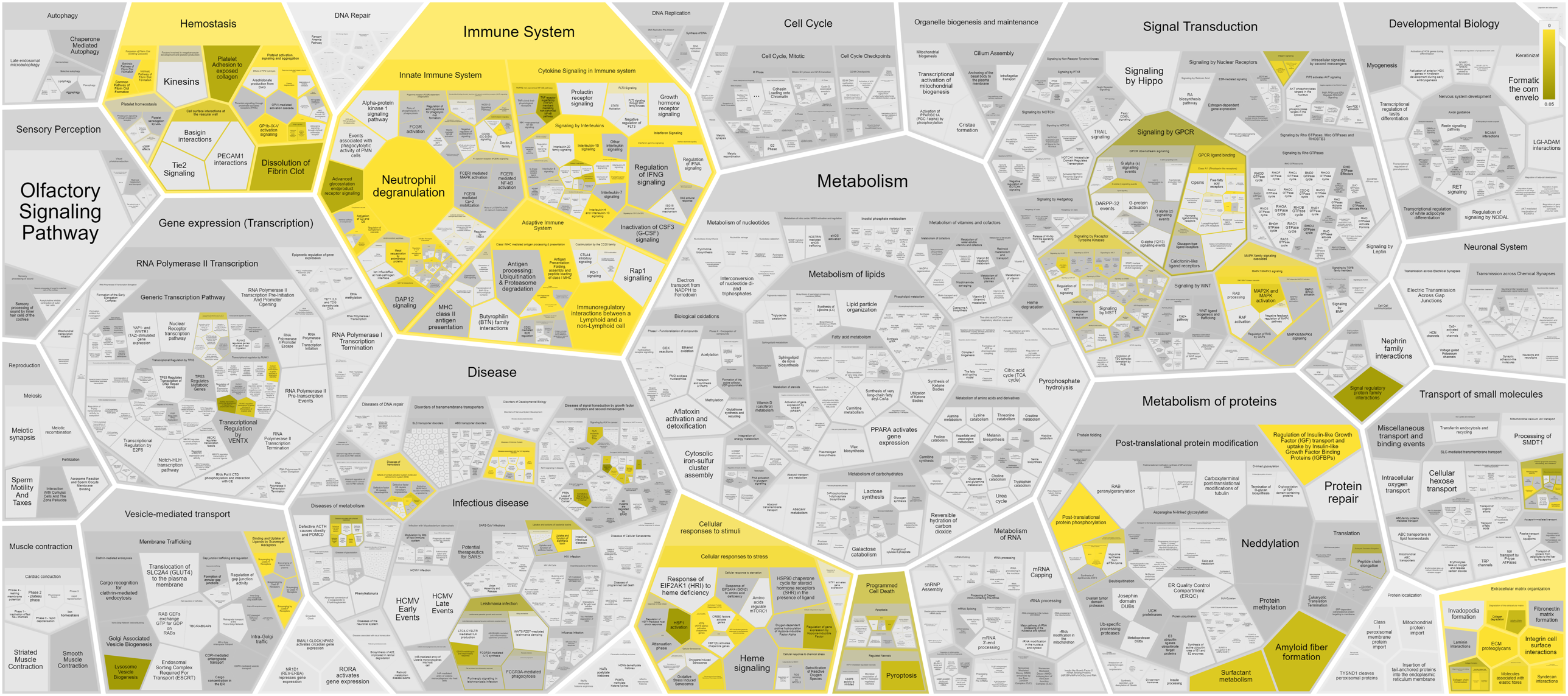

Supplement: Supplementary file 4 [file Image2.TIF]
