# Supplementary material for: Human Immune System Diseasome Networks and Female Oviductal Microenvironment: New Horizons to be Discovered
Source: Front Genet. 2022 Jan 27;12:795123. doi: 10.3389/fgene.2021.795123 (PMC8829125; doi:10.3389/fgene.2021.795123)
Supplement: Supplementary file 6 [file DataSheet1.PDF]

reactome

Pathway Analysis Report

This report contains the pathway analysis results for the submitted sample ''. Analysis was performed against Reactome version 77 on 11/10/2021. The web link to these results is:

https://reactome.org/PathwayBrowser/#/ANALYSIS=MjAyMTEwMTExMjU2MTdfNTM0MQ%3D%3D

Please keep in mind that analysis results are temporarily stored on our server. The storage period depends on usage of the service but is at least 7 days. As a result, please note that this URL is only valid for a limited time period and it might have expired.

Table of Contents

1. Introduction
2. Properties
3. Genome-wide overview
4. Most significant pathways
5. Pathways details
6. Identifiers found
7. Identifiers not found

1. Introduction

Reactome is a curated database of pathways and reactions in human biology. Reactions can be considered as pathway 'steps'. Reactome defines a 'reaction' as any event in biology that changes the state of a biological molecule. Binding, activation, translocation, degradation and classical biochemical events involving a catalyst are all reactions. Information in the database is authored by expert biologists, entered and maintained by Reactome's team of curators and editorial staff. Reactome content frequently cross-references other resources e.g. NCBI, Ensembl, UniProt, KEGG (Gene and Compound), ChEBI, PubMed and GO. Orthologous reactions inferred from annotation for Homo sapiens are available for 17 non-human species including mouse, rat, chicken, puffer fish, worm, fly, yeast, rice, and Arabidopsis. Pathways are represented by simple diagrams following an SBGN-like format.

Reactome's annotated data describe reactions possible if all annotated proteins and small molecules were present and active simultaneously in a cell. By overlaying an experimental dataset on these annotations, a user can perform a pathway over-representation analysis. By overlaying quantitative expression data or time series, a user can visualize the extent of change in affected pathways and its progression. A binomial test is used to calculate the probability shown for each result, and the p-values are corrected for the multiple testing (Benjamini–Hochberg procedure) that arises from evaluating the submitted list of identifiers against every pathway.

To learn more about our Pathway Analysis, please have a look at our relevant publications:

Fabregat A, Sidiropoulos K, Garapati P, Gillespie M, Hausmann K, Haw R, … D'Eustachio P (2016). The reactome pathway knowledgebase. Nucleic Acids Research, 44(D1), D481–D487. https://doi.org/10.1093/nar/gkv1351.

Fabregat A, Sidiropoulos K, Viteri G, Forner O, Marin-Garcia P, Arnau V, … Hermjakob H (2017). Reactome pathway analysis: a high-performance in-memory approach. BMC Bioinformatics, 18.

2. Properties

- This is an **overrepresentation** analysis: A statistical (hypergeometric distribution) test that determines whether certain Reactome pathways are over-represented (enriched) in the submitted data. It answers the question 'Does my list contain more proteins for pathway X than would be expected by chance?' This test produces a probability score, which is corrected for false discovery rate using the Benjamani-Hochberg method.
- 137 out of 145 identifiers in the sample were found in Reactome, where 828 pathways were hit by at least one of them.
- All non-human identifiers have been converted to their human equivalent.
- This report is filtered to show only results for species 'Homo sapiens' and resource 'all resources'.
- The unique ID for this analysis (token) is MjAyMTEwMTExMjU2MTdfNTM0MQ%3D%3D. This ID is valid for at least 7 days in Reactome's server. Use it to access Reactome services with your data.

3. Genome-wide overview

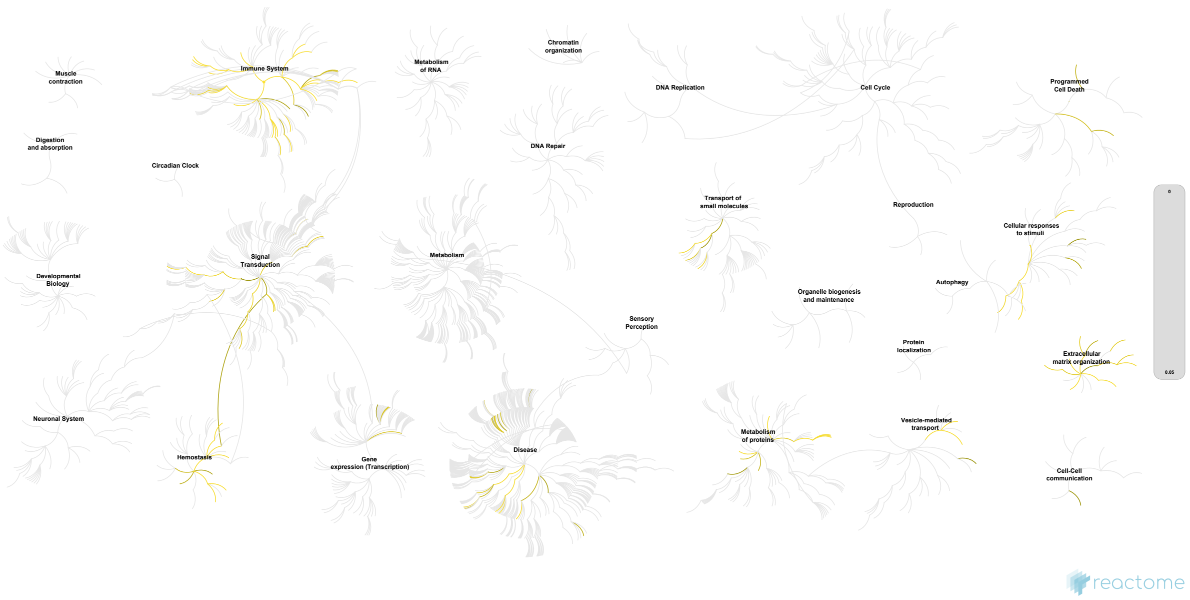

This figure shows a genome-wide overview of the results of your pathway analysis. Reactome pathways are arranged in a hierarchy. The center of each of the circular "bursts" is the root of one top-level pathway, for example "DNA Repair". Each step away from the center represents the next level lower in the pathway hierarchy. The color code denotes over-representation of that pathway in your input dataset. Light grey signifies pathways which are not significantly over-represented.

4. Most significant pathways

The following table shows the 25 most relevant pathways sorted by p-value.

Pathway name	Entities				Reactions	
	found	ratio	p-value	FDR*	found	ratio
Antigen Presentation: Folding, assembly and peptide loading of class I MHC	54 / 102	0.007	1.11e-16	5.66e-15	13 / 16	0.001
ER-Phagosome pathway	59 / 173	0.012	1.11e-16	5.66e-15	8 / 10	7.40e-04
Interleukin-10 signaling	27 / 86	0.006	1.11e-16	5.66e-15	12 / 15	0.001
Endosomal/Vacuolar pathway	51 / 82	0.006	1.11e-16	5.66e-15	3 / 4	2.96e-04
Interferon gamma signaling	56 / 250	0.017	1.11e-16	5.66e-15	12 / 16	0.001
Class I MHC mediated antigen processing & presentation	64 / 473	0.033	1.11e-16	5.66e-15	32 / 48	0.004
Platelet degranulation	25 / 139	0.01	1.11e-16	5.66e-15	6 / 11	8.14e-04
Antigen processing-Cross presentation	59 / 195	0.013	1.11e-16	5.66e-15	12 / 23	0.002
Interleukin-4 and Interleukin-13 signaling	35 / 211	0.015	1.11e-16	5.66e-15	21 / 47	0.003
Immunoregulatory interactions between a Lymphoid and a non-Lymphoid cell	57 / 316	0.022	1.11e-16	5.66e-15	19 / 44	0.003
Response to elevated platelet cytosolic Ca2+	25 / 146	0.01	1.11e-16	5.66e-15	6 / 14	0.001
Adaptive Immune System	75 / 1,004	0.069	1.11e-16	5.66e-15	76 / 264	0.02
Immune System	178 / 2,681	0.184	1.11e-16	5.66e-15	427 / 1,623	0.12
Signaling by Interleukins	64 / 643	0.044	1.11e-16	5.66e-15	115 / 493	0.036
Cytokine Signaling in Immune system	123 / 1,092	0.075	1.11e-16	5.66e-15	164 / 708	0.052
Interferon Signaling	57 / 394	0.027	1.11e-16	5.66e-15	15 / 69	0.005
Interferon alpha/beta signaling	53 / 186	0.013	1.11e-16	5.66e-15	2 / 22	0.002
Platelet activation, signaling and aggregation	29 / 291	0.02	2.17e-13	1.04e-11	37 / 116	0.009
Innate Immune System	63 / 1,334	0.092	1.47e-12	6.75e-11	195 / 710	0.053
Regulation of Insulin-like Growth Factor (IGF) transport and uptake by Insulin-like Growth Factor Binding Proteins (IGFBPs)	17 / 127	0.009	3.18e-10	1.37e-08	3 / 14	0.001
Post-translational protein phosphorylation	15 / 109	0.007	2.48e-09	1.02e-07	1 / 1	7.40e-05
Activation of C3 and C5	6 / 7	4.81e-04	4.97e-09	1.94e-07	3 / 3	2.22e-04
Regulation of Complement cascade	16 / 139	0.01	8.72e-09	3.31e-07	40 / 42	0.003

Pathway name	Entities				Reactions	
	found	ratio	p-value	FDR*	found	ratio
Neutrophil degranulation	29 / 480	0.033	2.16e-08	7.77e-07	7 / 10	7.40e-04
Hemostasis	39 / 801	0.055	2.41e-08	8.45e-07	108 / 334	0.025

* False Discovery Rate

5. Pathways details

For every pathway of the most significant pathways, we present its diagram, as well as a short summary, its bibliography and the list of inputs found in it.

1. Antigen Presentation: Folding, assembly and peptide loading of class I MHC (R-HSA-983170)

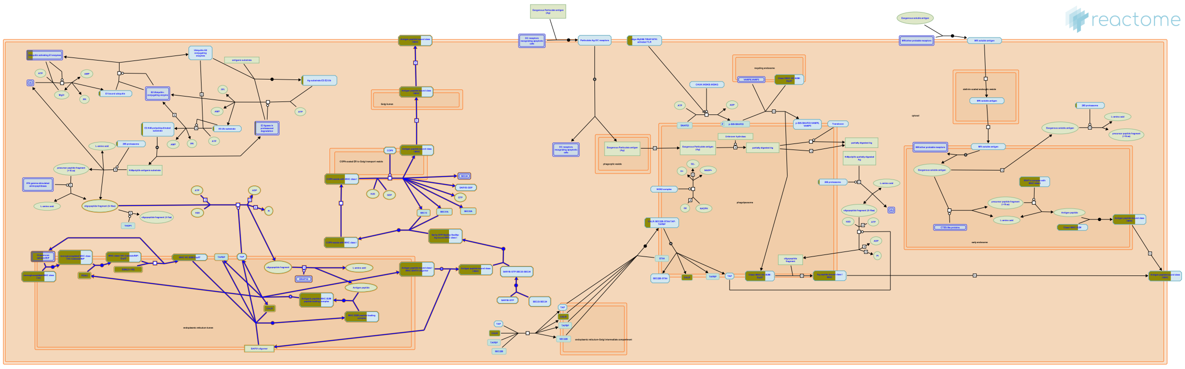

Unlike other glycoproteins, correct folding of MHC class I molecules is not sufficient to trigger their exit from the ER, they exit only after peptide loading. Described here is the process of antigen presentation which consists of the folding, assembly, and peptide loading of MHC class I molecules. The newly synthesized MHC class I Heavy Chain (HC) is initially folded with the help of several chaperones (calnexin, BiP, ERp57) and then binds with Beta-2-microglobulin (B2M). This MHC:B2M heterodimer enters the peptide loading complex (PLC), a multiprotein complex that includes calreticulin, endoplasmic reticulum resident protein 57 (ERp57), transporter associated with antigen processing (TAP) and tapasin. Peptides generated from Ub-proteolysis are transported into the ER through TAP. These peptides are further trimmed by ER-associated aminopeptidase (ERAP) and loaded on to MHC class I molecules. Stable MHC class I trimers with high-affinity peptide are transported from the ER to the cell surface by the Golgi apparatus.

References

Vyas JM, Van der Veen AG & Ploegh HL (2008). The known unknowns of antigen processing and presentation. Nat Rev Immunol, 8, 607-18.

Rock KL, York IA & Goldberg AL (2004). Post-proteasomal antigen processing for major histocompatibility complex class I presentation. Nat Immunol, 5, 670-7.

Kim Y, Kang K, Kim I, Lee YJ, Oh C, Ryoo J, ... Ahn K (2009). Molecular mechanisms of MHC class I-antigen processing: redox considerations. Antioxid Redox Signal, 11, 907-36.

Antoniou AN, Powis SJ & Elliott T (2003). Assembly and export of MHC class I peptide ligands. Curr Opin Immunol, 15, 75-81.

Wearsch PA & Cresswell P (2008). The quality control of MHC class I peptide loading. Curr Opin Cell Biol, 20, 624-31.

Edit history

Date	Action	Author
2010-10-29	Edited	Garapati P V

Date	Action	Author
2010-10-29	Authored	Garapati P V
2010-10-29	Created	Garapati P V
2011-02-11	Reviewed	Elliott T
2021-05-22	Modified	Shorser S

Entities found in this pathway (7)

Input	UniProt Id	Input	UniProt Id	Input	UniProt Id
B2M	P61769	CALR	P27797	HLA-B	P01889, P03989, P10319, P18463, P18464, P18465, P30460, P30461, P30462, P30464, P30466, P30475, P30479, P30480, P30481, P30483, P30484, P30485, P30486, P30487, P30488, P30490, P30491, P30492, P30493, P30495, P30498, P30685, Q04826, Q29718, Q29836, Q29940, Q31610, Q31612, Q95365
HLA-C	P04222, P10321, P30499, P30501, P30504, P30505, P30508, P30510, Q07000, Q29865, Q29960, Q29963, Q95604, Q9TNN7	HLA-G	P17693	HSPA5	P11021
PDIA3	P30101				

2. ER-Phagosome pathway (R-HSA-1236974)

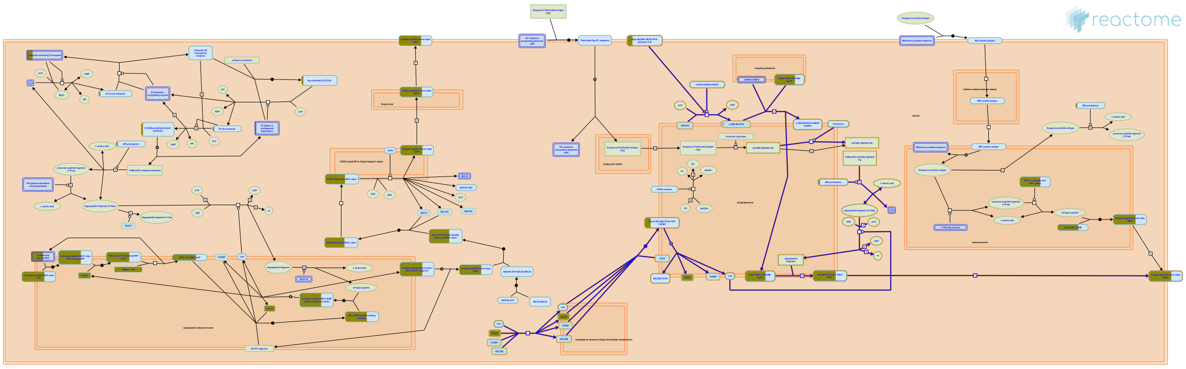

The other TAP-dependent cross-presentation mechanism in phagocytes is the endoplasmic reticulum (ER)-phagosome model. Desjardins proposed that ER is recruited to the cell surface, where it fuses with the plasma membrane, underneath phagocytic cups, to supply membrane for the formation of nascent phagosomes (Gagnon et al. 2002). Three independent studies simultaneously showed that ER contributes to the vast majority of phagosome membrane (Guermonprez et al. 2003, Houde et al. 2003, Ackerman et al. 2003). The composition of early phagosome membrane contains ER-resident proteins, the components required for cross-presentation. This model is similar to the phagosome-to-cytosol model in that Ag is translocated to cytosol for proteasomal degradation, but differs in that antigenic peptides are translocated back into the phagosome (instead of ER) for peptide:MHC-I complexes. ER fusion with phagosome introduces molecules that are involved in Ag transport to cytosol (Sec61) and proteasome-generated peptides back into the phagosome (TAP) for loading onto MHC-I.

Although the ER-phagosome pathway is controversial, the concept remains attractive as it explains how peptide-receptive MHC-I molecules could intersect with a relatively high concentration of exogenous antigens, presumably a crucial prerequisite for efficient cross-presentation (Basha et al. 2008).

References

Ackerman AL, Kyritsis C, Tampé R & Cresswell P (2003). Early phagosomes in dendritic cells form a cellular compartment sufficient for cross presentation of exogenous antigens. Proc Natl Acad Sci U S A, 100, 12889-94.

Guermonprez P, Saveanu L, Kleijmeer M, Davoust J, Van Endert P & Amigorena S (2003). ER-phagosome fusion defines an MHC class I cross-presentation compartment in dendritic cells. Nature, 425, 397-402.

Houde M, Bertholet S, Gagnon E, Brunet S, Goyette G, Laplante A, ... Desjardins M (2003). Phagosomes are competent organelles for antigen cross-presentation. Nature, 425, 402-6.

Gagnon E, Duclos S, Rondeau C, Chevet E, Cameron PH, Steele-Mortimer O, ... Desjardins M (2002). Endoplasmic reticulum-mediated phagocytosis is a mechanism of entry into macrophages. Cell, 110, 119-31.

Amigorena S & Savina A (2010). Intracellular mechanisms of antigen cross presentation in dendritic cells. Curr Opin Immunol, 22, 109-17.

Edit history

Date	Action	Author
2011-03-28	Edited	Garapati P V
2011-03-28	Authored	Garapati P V
2011-03-28	Created	Garapati P V
2011-05-13	Reviewed	Desjardins M, English L
2016-05-16	Reviewed	Bergeron JJ
2021-05-21	Modified	Shorser S

Entities found in this pathway (11)

Input	UniProt Id	Input	UniProt Id	Input	UniProt Id
B2M	P61769	CALR	P27797	FGA	P02671
FGG	P02679	HLA-B	P01889, P03989, P10319, P18463, P18464, P18465, P30460, P30461, P30462, P30464, P30466, P30475, P30479, P30480, P30481, P30483, P30484, P30485, P30486, P30487, P30488, P30490, P30491, P30492, P30493, P30495, P30498, P30685, Q04826, Q29718, Q29836, Q29940, Q31610, Q31612, Q95365	HLA-C	P04222, P10321, P30499, P30501, P30504, P30505, P30508, P30510, Q07000, Q29865, Q29960, Q29963, Q95604, Q9TNN7
HLA-G	P17693	HMGB1	P09429	PDIA3	P30101
PSMA7	O14818, Q8TAA3	S100A9	P06702		

3. Interleukin-10 signaling (R-HSA-6783783)

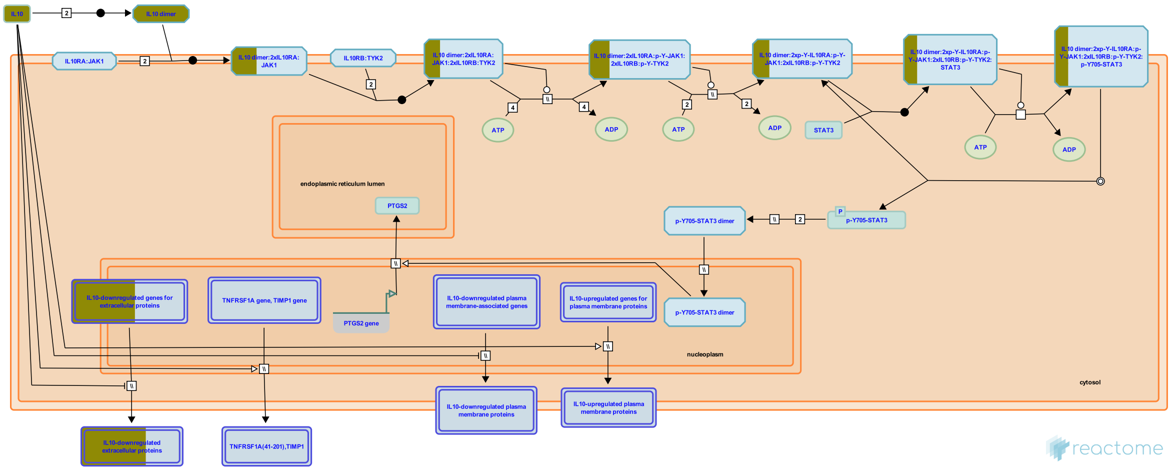

Interleukin-10 (IL10) was originally described as a factor named cytokine synthesis inhibitory factor that inhibited T-helper (Th) 1 activation and Th1 cytokine production (Fiorentino et al. 1989). It was found to be expressed by a variety of cell types including macrophages, dendritic cell subsets, B cells, several T-cell subpopulations including Th2 and T-regulatory cells (Tregs) and Natural Killer (NK) cells (Moore et al. 2001). It is now recognized that the biological effects of IL10 are directed at antigen-presenting cells (APCs) such as macrophages and dendritic cells (DCs), its effects on T-cell development and differentiation are largely indirect via inhibition of macrophage/dendritic cell activation and maturation (Pestka et al. 2004, Mocellin et al. 2004). T cells are thought to be the main source of IL10 (Hedrich & Bream 2010). IL10 inhibits a broad spectrum of activated macrophage/monocyte functions including monokine synthesis, NO production, and expression of class II MHC and costimulatory molecules such as IL12 and CD80/CD86 (de Waal Malefyt et al. 1991, Gazzinelli et al. 1992). Studies with recombinant cytokine and neutralizing antibodies revealed pleiotropic activities of IL10 on B, T, and mast cells (de Waal Malefyt et al. 1993, Rousset et al. 1992, Thompson-Snipes et al. 1991) and provided evidence for the in vivo significance of IL10 activities (Ishida et al. 1992, 1993). IL10 antagonizes the expression of MHC class II and the co-stimulatory molecules CD80/CD86 as well as the pro-inflammatory cytokines IL1Beta, IL6, IL8, TNFalpha and especially IL12 (Fiorentino et al. 1991, D'Andrea et al. 1993). The biological role of IL10 is not limited to inactivation of APCs, it also enhances B cell, granulocyte, mast cell, and keratinocyte growth/differentiation, as well as NK-cell and CD8+ cytotoxic T-cell activation (Moore et al. 2001, Hedrich & Bream 2010). IL10 also enhances NK-cell proliferation and/or production of IFN-gamma (Cai et al. 1999).

IL10-deficient mice exhibited inflammatory bowel disease (IBD) and other exaggerated inflammatory responses (Kuhn et al. 1993, Berg et al. 1995) indicating a critical role for IL10 in limiting inflammatory responses. Dysregulation of IL10 is linked with susceptibility to numerous infectious and autoimmune diseases in humans and mouse models (Hedrich & Bream 2010).

IL10 signaling is initiated by binding of homodimeric IL10 to the extracellular domains of two adjoining IL10RA molecules. This tetramer then binds two IL10RB chains. IL10RB cannot bind to IL10 unless bound to IL10RA (Ding et al. 2001, Yoon et al. 2006); binding of IL10 to IL10RA without the co-presence of IL10RB fails to initiate signal transduction (Kotenko et al. 1997).

IL10 binding activates the receptor-associated Janus tyrosine kinases, JAK1 and TYK2, which are constitutively bound to IL10R1 and IL10R2 respectively. In the classic model of receptor activation assembly of the receptor complex is believed to enable JAK1/TYK2 to phosphorylate and activate each other. Alternatively the binding of IL10 may cause conformational changes that allow the pseudokinase inhibitory domain of one JAK kinase to move away from the kinase domain of the other JAK within the receptor dimer-JAK complex, allowing the two kinase domains to interact and trans-activate (Waters & Brooks 2015).

The activated JAK kinases phosphorylate the intracellular domains of the IL10R1 chains on specific tyrosine residues. These phosphorylated tyrosine residues and their flanking peptide sequences serve as temporary docking sites for the latent, cytosolic, transcription factor, STAT3. STAT3 transiently docks on the IL10R1 chain via its SH2 domain, and is in turn tyrosine phosphorylated by the receptor-associated JAKs. Once activated, it dissociates from the receptor, dimerizes with other STAT3 molecules, and translocates to the nucleus where it binds with high affinity to STAT-binding elements (SBEs) in the promoters of IL-10-inducible genes (Donnelly et al. 1999).

References

Moore KW, de Waal Malefyt R, Coffman RL & O'Garra A (2001). Interleukin-10 and the interleukin-10 receptor. Annu. Rev. Immunol., 19, 683-765.

Edit history

Date	Action	Author
2015-06-17	Authored	Jupe S
2015-06-17	Created	Jupe S
2016-09-05	Reviewed	Meldal BH
2016-11-14	Edited	Jupe S
2021-05-31	Modified	Shorser S

Entities found in this pathway (13)

Input	UniProt Id
CCL2	P13500
CCL5	P13501
CXCL1	P09341
IL10	P22301
TNF	P01375

Input	UniProt Id
CCL3L3	P16619
CSF2	P04141
CXCL2	P19875
IL1B	P01583, P01584

Input	UniProt Id
CCL4	O00626, P13236
CSF3	P09919
CXCL8	P09341, P10145
IL6	P05231

Input	Ensembl Id
CCL2	ENSG00000108691
CCL5	ENSG00000271503
CXCL1	ENSG00000163739
IL1B	ENSG00000125538

Input	Ensembl Id
CCL3L3	ENSG00000277768
CSF2	ENSG00000164400
CXCL2	ENSG00000081041
IL6	ENSG00000136244

Input	Ensembl Id
CCL4	ENSG00000275302
CSF3	ENSG00000108342
CXCL8	ENSG00000169429
TNF	ENSG00000232810

4. Endosomal/Vacuolar pathway (R-HSA-1236977)

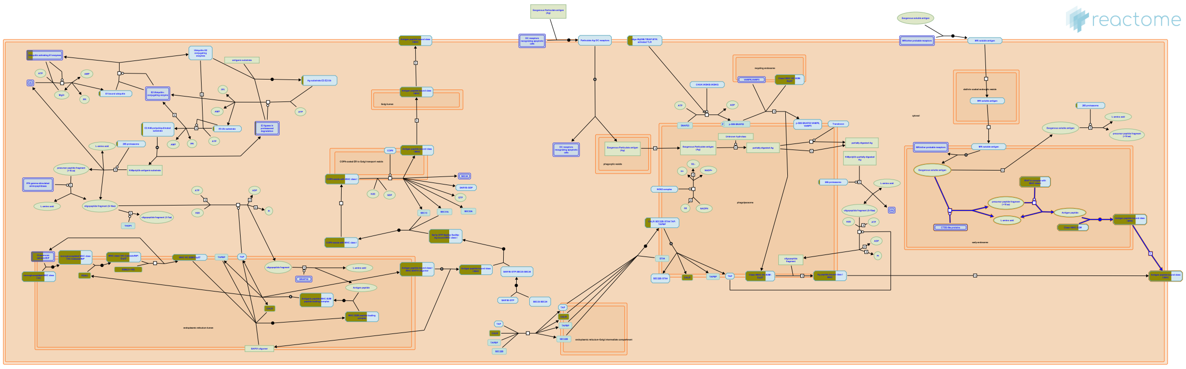

Cellular compartments: early endosome.

Some antigens are cross-presented through a vacuolar mechanism that involves generation of antigenic peptides and their loading on to MHC-I molecules within the endosomal compartment in a proteasome and TAP-independent manner. Antigens within the endosome are processed by cathepsin S and other proteases into antigenic peptides. Loading of these peptides onto MHC-I molecules occurs directly within early and late endosomal compartments. Why certain antigens are cross-presented exclusively by the cytosolic pathway while others use the vacuolar pathway is unknown. It may be because some epitopes cannot be generated by endosomal proteolysis, or are completely destroyed. Alternatively, the physical form of the antigen may influence its accessibility to the endosomal or vacuolar pathways (Shen et al. 2004).

References

Di Pucchio T, Chatterjee B, Smed-Sörensen A, Clayton S, Palazzo A, Montes M, ... Connolly JE (2008). Direct proteasome-independent cross-presentation of viral antigen by plasmacytoid dendritic cells on major histocompatibility complex class I. Nat Immunol, 9, 551-7.

Edit history

Date	Action	Author
2011-03-28	Edited	Garapati P V
2011-03-28	Authored	Garapati P V
2011-03-28	Created	Garapati P V
2011-05-13	Reviewed	Desjardins M, English L
2021-05-21	Modified	Shorser S

Entities found in this pathway (4)

Input	UniProt Id	Input	UniProt Id
B2M	P61769	HLA-B	P01889, P03989, P10319, P18463, P18464, P18465, P30460, P30461, P30462, P30464, P30466, P30475, P30479, P30480, P30481, P30483, P30484, P30485, P30486, P30487, P30488, P30490, P30491, P30492, P30493, P30495, P30498, P30685, Q04826, Q29718, Q29836, Q29940, Q31610, Q31612, Q95365
HLA-C	P04222, P10321, P30499, P30501, P30504, P30505, P30508, P30510, Q07000, Q29865, Q29960, Q29963, Q95604, Q9TNN7	HLA-G	P17693

5. Interferon gamma signaling (R-HSA-877300)

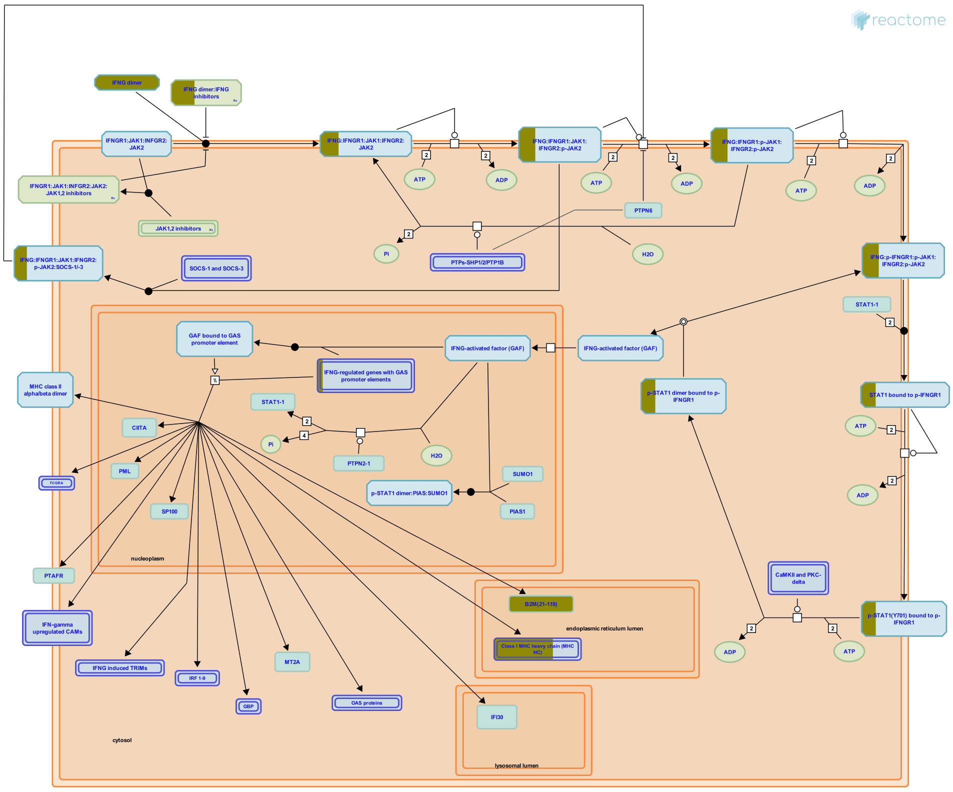

Interferon-gamma (IFN-gamma) belongs to the type II interferon family and is secreted by activated immune cells-primarily T and NK cells, but also B-cells and APC. INFG exerts its effect on cells by interacting with the specific IFN-gamma receptor (IFNGR). IFNGR consists of two chains, namely IFNGR1 (also known as the IFNGR alpha chain) and IFNGR2 (also known as the IFNGR beta chain). IFNGR1 is the ligand binding receptor and is required but not sufficient for signal transduction, whereas IFNGR2 do not bind IFNG independently but mainly plays a role in IFNG signaling and is generally the limiting factor in IFNG responsiveness. Both IFNGR chains lack intrinsic kinase/phosphatase activity and thus rely on other signaling proteins like Janus-activated kinase 1 (JAK1), JAK2 and Signal transducer and activator of transcription 1 (STAT-1) for signal transduction. IFNGR complex in its resting state is a preformed tetramer and upon IFNG association undergoes a conformational change. This conformational change induces the phosphorylation and activation of JAK1, JAK2, and STAT1 which in turn induces genes containing the gamma-interferon activation sequence (GAS) in the promoter.

References

Gough DJ, Levy DE, Johnstone RW & Clarke CJ (2008). IFNgamma signaling-does it mean JAK-STAT?. Cytokine Growth Factor Rev, 19, 383-94.

Pestka S, Kotenko SV, Muthukumaran G, Izotova LS, Cook JR & Garotta G (1997). The interferon gamma (IFN-gamma) receptor: a paradigm for the multichain cytokine receptor. Cytokine Growth Factor Rev, 8, 189-206.

Bach EA, Aguet M & Schreiber RD (1997). The IFN gamma receptor: a paradigm for cytokine receptor signaling. Annu Rev Immunol, 15, 563-91.

Schroder K, Hertzog PJ, Ravasi T & Hume DA (2004). Interferon-gamma: an overview of signals, mechanisms and functions. J Leukoc Biol, 75, 163-89.

Edit history

Date	Action	Author
2010-06-08	Edited	Garapati P V
2010-06-08	Authored	Garapati P V
2010-06-11	Created	Garapati P V
2010-08-17	Reviewed	Abdul-Sater AA, Schindler C
2021-05-31	Modified	Shorser S

Entities found in this pathway (5)

Input	UniProt Id	Input	UniProt Id	Input	UniProt Id
B2M	P61769	HLA-B	P01889, P03989, P10319, P18463, P18464, P18465, P30460, P30461, P30462, P30464, P30466, P30475, P30479, P30480, P30481, P30483, P30484, P30485, P30486, P30487, P30488, P30490, P30491, P30492, P30493, P30495, P30498, P30685, Q04826, Q29718, Q29836, Q29940, Q31610, Q31612, Q95365	HLA-C	P04222, P10321, P30499, P30501, P30504, P30505, P30508, P30510, Q07000, Q29865, Q29960, Q29963, Q95604, Q9TNN7
HLA-G	P17693	IFNG	P01579		

Input	Ensembl Id	Input	Ensembl Id
B2M	ENSG00000166710	HLA-B	ENSG00000234745
HLA-C	ENSG00000204525	HLA-G	ENSG00000204632

6. Class I MHC mediated antigen processing & presentation (R-HSA-983169)

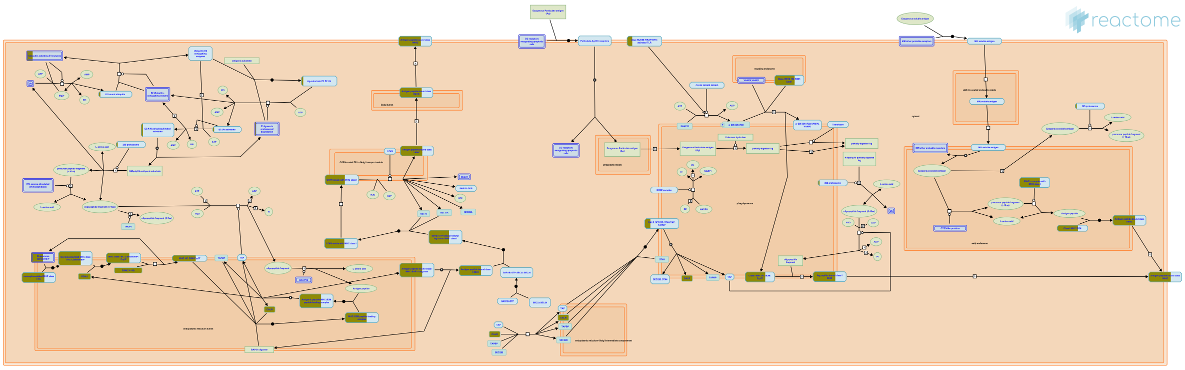

Major histocompatibility complex (MHC) class I molecules play an important role in cell mediated immunity by reporting on intracellular events such as viral infection, the presence of intracellular bacteria or tumor-associated antigens. They bind peptide fragments of these proteins and presenting them to CD8+ T cells at the cell surface. This enables cytotoxic T cells to identify and eliminate cells that are synthesizing abnormal or foreign proteins. MHC class I is a trimeric complex composed of a polymorphic heavy chain (HC or alpha chain) and an invariable light chain, known as beta2-microglobulin (B2M) plus an 8-10 residue peptide ligand. Represented here are the events in the biosynthesis of MHC class I molecules, including generation of antigenic peptides by the ubiquitin/26S-proteasome system, delivery of these peptides to the endoplasmic reticulum (ER), loading of peptides to MHC class I molecules and display of MHC class I complexes on the cell surface.

References

York IA & Rock KL (1996). Antigen processing and presentation by the class I major histocompatibility complex. Annu Rev Immunol, 14, 369-96.

Vyas JM, Van der Veen AG & Ploegh HL (2008). The known unknowns of antigen processing and presentation. Nat Rev Immunol, 8, 607-18.

Purcell AW & Elliott T (2008). Molecular machinations of the MHC-I peptide loading complex. Curr Opin Immunol, 20, 75-81.

Wearsch PA & Cresswell P (2008). The quality control of MHC class I peptide loading. Curr Opin Cell Biol, 20, 624-31.

Elliott T & Neefjes J (2006). The complex route to MHC class I-peptide complexes. Cell, 127, 249-51.

Edit history

Date	Action	Author
2010-10-29	Edited	Garapati P V
2010-10-29	Authored	Garapati P V
2010-10-29	Created	Garapati P V
2011-02-11	Reviewed	Elliott T
2021-05-22	Modified	Shorser S

Entities found in this pathway (15)

Input	UniProt Id	Input	UniProt Id	Input	UniProt Id
B2M	P61769	CALR	P27797	CDH1	Q9UM11
FGA	P02671	FGG	P02679	HLA-B	P01889, P03989, P10319, P18463, P18464, P18465, P30460, P30461, P30462, P30464, P30466, P30475, P30479, P30480, P30481, P30483, P30484, P30485, P30486, P30487, P30488, P30490, P30491, P30492, P30493, P30495, P30498, P30685, Q04826, Q29718, Q29836, Q29940, Q31610, Q31612, Q95365
HLA-C	P04222, P10321, P30499, P30501, P30504, P30505, P30508, P30510, Q07000, Q29865, Q29960, Q29963, Q95604, Q9TNN7	HLA-G	P17693	HMGB1	P09429
HSPA5	P11021	HUWE1	Q7Z6Z7, Q8IYU2	PDIA3	P30101
PSMA7	O14818, Q8TAA3	S100A9	P06702	UBA3	Q8TBC4

7. Platelet degranulation (R-HSA-114608)

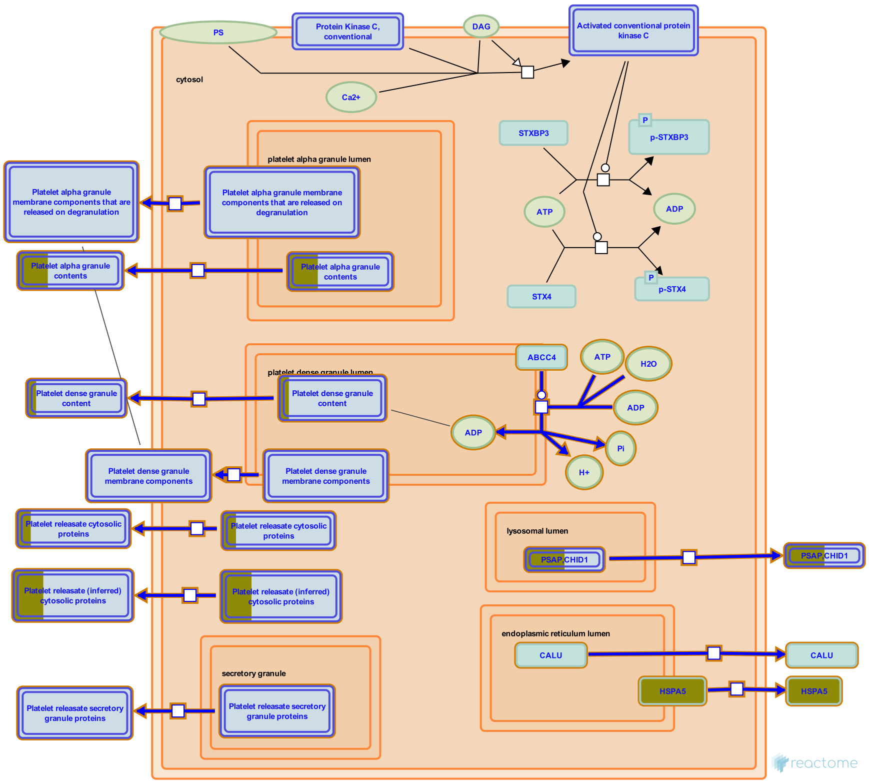

Platelets function as exocytotic cells, secreting a plethora of effector molecules at sites of vascular injury. Platelets contain a number of distinguishable storage granules including alpha granules, dense granules and lysosomes. On activation platelets release a variety of proteins, largely from storage granules but also as the result of apparent cell lysis. These act in an autocrine or paracrine fashion to modulate cell signaling.

Alpha granules contain mainly polypeptides such as fibrinogen, von Willebrand factor, growth factors and protease inhibitors that that supplement thrombin generation at the site of injury. Dense granules contain small molecules, particularly adenosine diphosphate (ADP), adenosine triphosphate (ATP), serotonin and calcium, all recruit platelets to the site of injury.

The molecular mechanism which facilitates granule release involves soluble NSF attachment protein receptors (SNAREs), which assemble into complexes to form a universal membrane fusion apparatus. Although all cells use SNAREs for membrane fusion, different cells possess different SNARE isoforms. Platelets and chromaffin cells use many of the same chaperone proteins to regulate SNARE-mediated secretion (Fitch-Tewfik & Flaumenhaft 2013).

References

Gresele P, Page CP, Fuster V & Vermylen J (2002). *Platelets in thrombotic and non-thrombotic disorders.* , 435-437.

Coppinger JA, Cagney G, Toomey S, Kislinger T, Belton O, McRedmond JP, ... Maguire PB (2004). Characterization of the proteins released from activated platelets leads to localization of novel platelet proteins in human atherosclerotic lesions. Blood, 103, 2096-104.

Edit history

Date	Action	Author
2004-09-25	Created	Farndale R, Pace NP, de Bono B
2021-05-22	Modified	Shorser S

Entities found in this pathway (23)

Input	UniProt Id	Input	UniProt Id	Input	UniProt Id
A1BG	P04217	A2M	P01023	AHSG	P02765
CAP1	Q01518	CLEC3B	P05452	FGA	P02671
FGG	P02679	FLNA	P21333	FN1	P02751
HGF	P14210	HSPA5	P11021	KNG1	P01042
LGALS3BP	Q08380	ORM2	P19652	PDGFB	P01127
PLG	P00747	PROS1	P07225	PSAP	P07602
SERPINA1	P01009, P01011	SERPING1	P05155	TGFB1	P01137
VCL	P18206	VEGFA	P15692, P49765		

8. Antigen processing-Cross presentation (R-HSA-1236975)

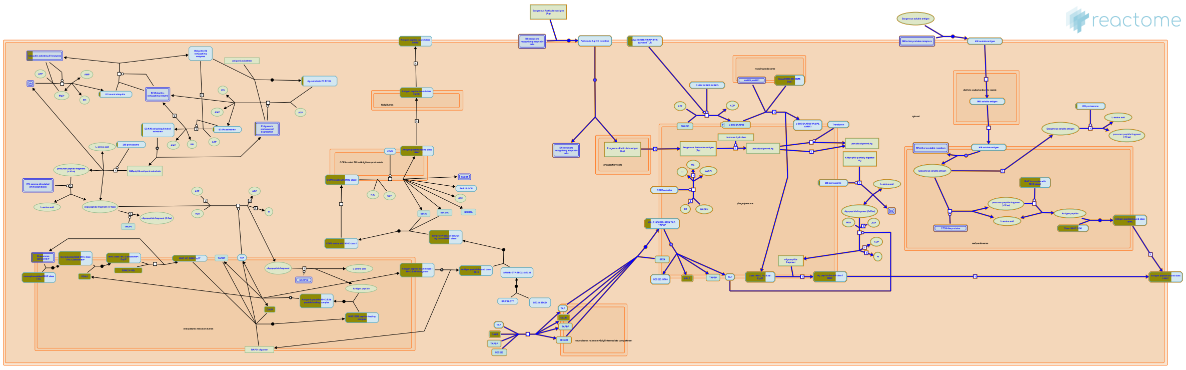

MHC class I molecules generally present peptide antigens derived from proteins synthesized by the cell itself to CD8+ T cells. However, in some circumstances, antigens from extracellular environment can be presented on MHC class I to stimulate CD8+ T cell immunity, a process termed cross-presentation (Rock & Shen. 2005). Cross-presentation/cross-priming is the ability of antigen presenting cells (APCs) to present exogenous antigens on MHC class I molecules to CD8+ T lymphocytes. Among all the APCs, Dendritic cells (DC) are the dominant antigen cross presenting cell types in vivo, although macrophages and B cells appear to cross present model antigens in vitro with a low degree of efficiency (Amigorena & Savina. 2010, Ackermann & Peter Cresswell. 2004). Compared to macrophages, DCs have low levels of lysosomal proteases and exhibit limited lysosomal degradation (Delamarre et al. 2005). This limited proteolysis of internalized antigens by DCs might contribute to their high efficiency for cross presentation (Monua & Trombetta. 2007). APCs acquire the exogenous antigens through endocytic mechanisms, especially phagosomes for particulate/cell-associated antigens and endosomes for soluble protein antigens. There does not seem to be a unique pathway for cross-presentation but rather different potential mechanisms of cross-presentation have been proposed. These proposed pathways can be classified according to the location where two key events occur: 1) processing of the antigenic protein and 2) loading of the processed peptide on to MHC I molecule (Blanchard & Shastri. 2010). Based on the requirement for TAP and cytosolic proteases two mechanisms have been described, a cytosolic pathway (TAP-dependent and proteasome-dependent) or a vacuolar pathway (TAP- and proteasome-independent) (Blanchard & Shastri. 2010, Amigorena & Savina. 2010). Regarding peptide-loading, MHC I could be loaded in the ER or in the phagosome and recycled to cell surface (Blanchard & Shastri. 2010). Exogenous soluble antigens are cross-presented by dendritic cells, albeit with lower efficiency than for particulate substrates. Soluble antigens destined for cross-presentation are taken up by distinct endocytosis mechanisms which route them into stable early endosomes and then to the cytoplasm for proteasomal degradation and peptide loading. The outcome of the cross presentation can be either tolerance or immunity (Rock & Shen. 2005).

References

Cresswell P, Ackerman AL, Giodini A, Peaper DR & Wearsch PA (2005). Mechanisms of MHC class I-restricted antigen processing and cross-presentation. Immunol Rev, 207, 145-57.

Monu N & Trombetta ES (2007). Cross-talk between the endocytic pathway and the endoplasmic reticulum in cross-presentation by MHC class I molecules. Curr Opin Immunol, 19, 66-72.

Amigorena S & Savina A (2010). Intracellular mechanisms of antigen cross presentation in dendritic cells. Curr Opin Immunol, 22, 109-17.

Delamarre L, Pack M, Chang H, Mellman I & Trombetta ES (2005). Differential lysosomal proteolysis in antigen-presenting cells determines antigen fate. Science, 307, 1630-4.

Giodini A, Rahner C & Cresswell P (2009). Receptor-mediated phagocytosis elicits cross-presentation in nonprofessional antigen-presenting cells. Proc Natl Acad Sci U S A, 106, 3324-9.

Edit history

Date	Action	Author
2011-03-28	Edited	Garapati P V
2011-03-28	Authored	Garapati P V
2011-03-28	Created	Garapati P V
2011-05-13	Reviewed	Desjardins M, English L
2021-05-22	Modified	Shorser S

Entities found in this pathway (11)

Input	UniProt Id	Input	UniProt Id	Input	UniProt Id
B2M	P61769	CALR	P27797	FGA	P02671
FGG	P02679	HLA-B	P01889, P03989, P10319, P18463, P18464, P18465, P30460, P30461, P30462, P30464, P30466, P30475, P30479, P30480, P30481, P30483, P30484, P30485, P30486, P30487, P30488, P30490, P30491, P30492, P30493, P30495, P30498, P30685, Q04826, Q29718, Q29836, Q29940, Q31610, Q31612, Q95365	HLA-C	P04222, P10321, P30499, P30501, P30504, P30505, P30508, P30510, Q07000, Q29865, Q29960, Q29963, Q95604, Q9TNN7
HLA-G	P17693	HMGB1	P09429	PDIA3	P30101
PSMA7	O14818, Q8TAA3	S100A9	P06702		

9. Interleukin-4 and Interleukin-13 signaling (R-HSA-6785807)

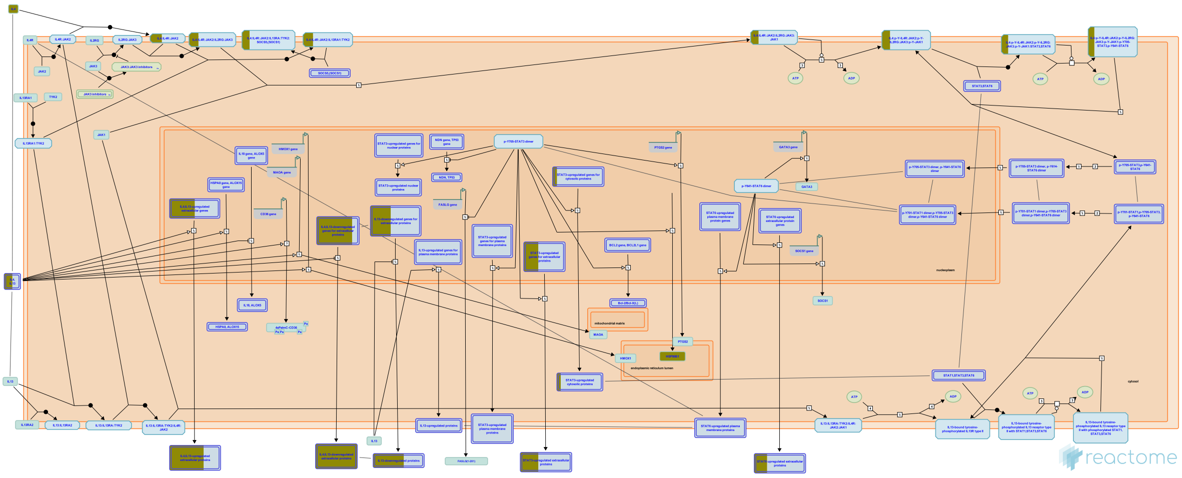

Interleukin-4 (IL4) is a principal regulatory cytokine during the immune response, crucially important in allergy and asthma (Nelms et al. 1999). When resting T cells are antigen-activated and expand in response to Interleukin-2 (IL2), they can differentiate as Type 1 (Th1) or Type 2 (Th2) T helper cells. The outcome is influenced by IL4. Th2 cells secrete IL4, which both stimulates Th2 in an autocrine fashion and acts as a potent B cell growth factor to promote humoral immunity (Nelms et al. 1999).

Interleukin-13 (IL13) is an immunoregulatory cytokine secreted predominantly by activated Th2 cells. It is a key mediator in the pathogenesis of allergic inflammation. IL13 shares many functional properties with IL4, stemming from the fact that they share a common receptor subunit. IL13 receptors are expressed on human B cells, basophils, eosinophils, mast cells, endothelial cells, fibroblasts, monocytes, macrophages, respiratory epithelial cells, and smooth muscle cells, but unlike IL4, not T cells. Thus IL13 does not appear to be important in the initial differentiation of CD4 T cells into Th2 cells, rather it is important in the effector phase of allergic inflammation (Hershey et al. 2003).

IL4 and IL13 induce "alternative activation" of macrophages, inducing an anti-inflammatory phenotype by signaling through IL4R alpha in a STAT6 dependent manner. This signaling plays an important role in the Th2 response, mediating anti-parasitic effects and aiding wound healing (Gordon & Martinez 2010, Loke et al. 2002)

There are two types of IL4 receptor complex (Andrews et al. 2006). Type I IL4R (IL4R1) is predominantly expressed on the surface of hematopoietic cells and consists of IL4R and IL2RG, the common gamma chain. Type II IL4R (IL4R2) is predominantly expressed on the surface of nonhematopoietic cells, it consists of IL4R and IL13RA1 and is also the type II receptor for IL13. (Obiri et al. 1995, Aman et al. 1996, Hilton et al. 1996, Miloux et al. 1997, Zhang et al. 1997). The second receptor for IL13 consists of IL4R and Interleukin-13 receptor alpha 2 (IL13RA2), sometimes called Interleukin-13 binding protein (IL13BP). It has a high affinity receptor for IL13 (Kd = 250 pmol/L) but is not sufficient to render cells responsive to IL13, even in the presence of IL4R (Donaldson et al. 1998). It is reported to exist in soluble form (Zhang et al. 1997) and when overexpressed reduces JAK-STAT signaling (Kawakami et al. 2001). It's function may be to prevent IL13 signalling via the functional IL4R:IL13RA1 receptor. IL13RA2 is overexpressed and enhances cell invasion in some human cancers (Joshi & Puri 2012).

The first step in the formation of IL4R1 (IL4:IL4R:IL2RB) is the binding of IL4 with IL4R (Hoffman et al. 1995, Shen et al. 1996, Hage et al. 1999). This is also the first step in formation of IL4R2 (IL4:IL4R:IL13RA1). After the initial binding of IL4 and IL4R, IL2RB binds (LaPorte et al. 2008), to form IL4R1. Alternatively, IL13RA1 binds, forming IL4R2. In contrast, the type II IL13 complex (IL13R2) forms with IL13 first binding to IL13RA1 followed by recruitment of IL4R (Wang et al. 2009).

Crystal structures of the IL4:IL4R:IL2RG, IL4:IL4R:IL13RA1 and IL13:IL4R:IL13RA1 complexes have been determined (LaPorte et al. 2008). Consistent with these structures, in monocytes IL4R is tyrosine phosphorylated in response to both IL4 and IL13 (Roy et al. 2002, Gordon & Martinez 2010) while IL13RA1 phosphorylation is induced only by IL13 (Roy et al. 2002, LaPorte et al. 2008) and IL2RG phosphorylation is induced only by IL4 (Roy et al. 2002).

Both IL4 receptor complexes signal through Jak/STAT cascades. IL4R is constitutively-associated with JAK2 (Roy et al. 2002) and associates with JAK1 following binding of IL4 (Yin et al. 1994) or IL13 (Roy et al. 2002). IL2RG constitutively associates with JAK3 (Boussiotis et al. 1994, Russell et al. 1994). IL13RA1 constitutively associates with TYK2 (Umeshita-Suyama et al. 2000, Roy et al. 2002, LaPorte et al. 2008, Bhattacharjee et al. 2013).

IL4 binding to IL4R1 leads to phosphorylation of JAK1 (but not JAK2) and STAT6 activation (Takeda et al. 1994, Ratthe et al. 2007, Bhattacharjee et al. 2013).

IL13 binding increases activating tyrosine-99 phosphorylation of IL13RA1 but not that of IL2RG. IL4 binding to IL2RG leads to its tyrosine phosphorylation (Roy et al. 2002). IL13 binding to IL4R2 leads to TYK2 and JAK2 (but not JAK1) phosphorylation (Roy & Cathcart 1998, Roy et al. 2002).

Phosphorylated TYK2 binds and phosphorylates STAT6 and possibly STAT1 (Bhattacharjee et al. 2013).

A second mechanism of signal transduction activated by IL4 and IL13 leads to the insulin receptor substrate (IRS) family (Kelly-Welch et al. 2003). IL4R1 associates with insulin receptor substrate 2 and activates the PI3K/Akt and Ras/MEK/Erk pathways involved in cell proliferation, survival and translational control. IL4R2 does not associate with insulin receptor substrate 2 and consequently the PI3K/Akt and Ras/MEK/Erk pathways are not activated (Busch-Dienstfertig & González-Rodríguez 2013).

References

Nelms K, Keegan AD, Zamorano J, Ryan JJ & Paul WE (1999). The IL-4 receptor: signaling mechanisms and biologic functions. Annu. Rev. Immunol., 17, 701-38.

Hershey GK (2003). IL-13 receptors and signaling pathways: an evolving web. J. Allergy Clin. Immunol., 111, 677-90; quiz 691.

Edit history

Date	Action	Author
2015-07-01	Authored	Jupe S
2015-07-01	Created	Jupe S
2016-09-02	Edited	Jupe S
2016-09-02	Reviewed	Leibovich SJ

Date	Action	Author
2021-05-31	Modified	Shorser S

Entities found in this pathway (18)

Input	UniProt Id	Input	UniProt Id	Input	UniProt Id
ANXA1	P04083	CCL2	P13500	CCL4	O00626, P51671
COL1A2	P08123	CXCL8	P10145	FN1	P02751
HGF	P14210	HSP90AA1	P07900	HSP90B1	P14625
IL10	P22301	IL1B	P01583, P01584	IL4	P05112
IL6	P05231	LCN2	P80188	OSM	P13725
TGFB1	P01137	TNF	P01375	VEGFA	P15692

Input	Ensembl Id	Input	Ensembl Id	Input	Ensembl Id
ANXA1	ENSG00000135046	CCL2	ENSG00000108691	COL1A2	ENSG00000164692
CXCL8	ENSG00000169429	FN1	ENSG00000115414	HGF	ENSG00000019991
HSP90AA1	ENSG00000080824	IL10	ENSG00000136634	IL1B	ENSG00000125538
IL6	ENSG00000136244	LCN2	ENSG00000148346	OSM	ENSG00000099985
TGFB1	ENSG00000105329	TNF	ENSG00000232810	VEGFA	ENSG00000112715

10. Immunoregulatory interactions between a Lymphoid and a non-Lymphoid cell (R-HSA-198933)

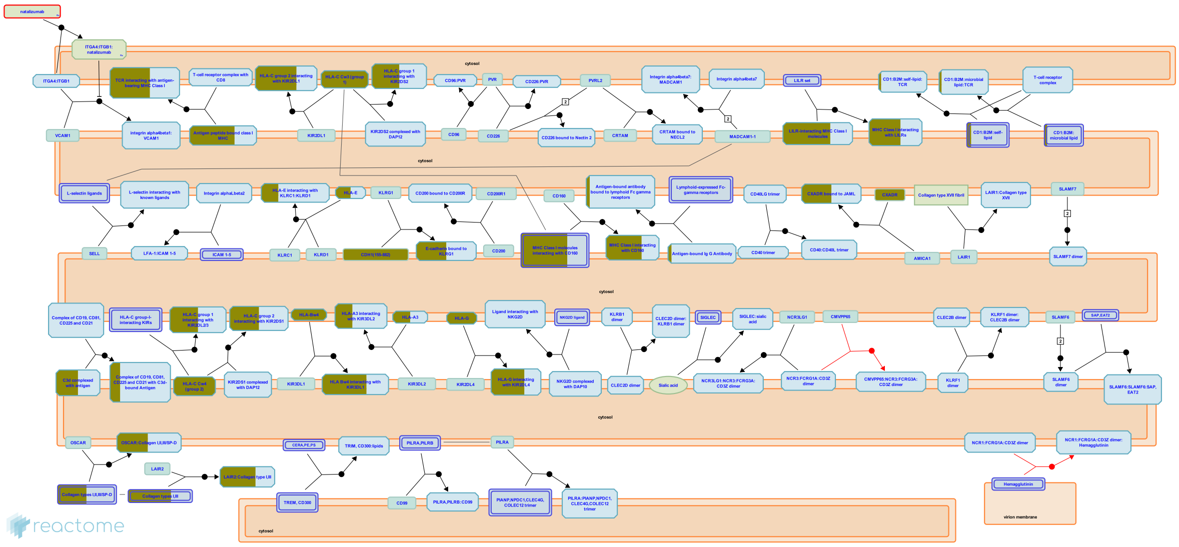

A number of receptors and cell adhesion molecules play a key role in modifying the response of cells of lymphoid origin (such as B-, T- and NK cells) to self and tumor antigens, as well as to pathogenic organisms.

Molecules such as KIRs and LILRs form part of a crucial surveillance system that looks out for any derangement, usually caused by cancer or viral infection, in MHC Class I presentation. Somatic cells are also able to report internal functional impairment by displaying surface stress markers such as MICA. The presence of these molecules on somatic cells is picked up by C-lectin NK immune receptors.

Lymphoid cells are able to regulate their location and movement in accordance to their state of activation, and home in on tissues expressing the appropriate complementary ligands. For example, lymphoid cells may fine tune the presence and concentration of adhesion molecules belonging to the IgSF, Selectin and Integrin class that interact with a number of vascular markers of inflammation.

Furthermore, there are a number of avenues through which lymphoid cells may interact with antigen. This may be presented directly to a specific T-cell receptor in the context of an MHC molecule. Antigen-antibody complexes may anchor to the cell via a small number of lymphoid-specific Fc receptors that may, in turn, influence cell function further. Activated complement factor C3d binds to both antigen and to cell surface receptor CD21. In such cases, the far-reaching influence of CD19 on B-lymphocyte function is tempered by its interaction with CD21.

References

Carrasco YR & Batista FD (2006). B cell recognition of membrane-bound antigen: an exquisite way of sensing ligands. Curr Opin Immunol, 18, 286-91.

Cemerski S & Shaw A (2006). Immune synapses in T-cell activation. Curr Opin Immunol, 18, 298-304 .

Nedvetzki S, Sowinski S, Eagle RA, Harris J, Vely F, Pende D, ... Davis DM (2007). Reciprocal regulation of human natural killer cells and macrophages associated with distinct immune synapses. Blood, 109, 3776-85.

Bromley SK, Burack WR, Johnson KG, Somersalo K, Sims TN, Sumen C, ... Dustin ML (2001). The immunological synapse. Annu Rev Immunol, 19, 375-96.

Vivier E, Tomasello E, Baratin M, Walzer T & Ugolini S (2008). Functions of natural killer cells. Nat. Immunol., 9, 503-10.

Edit history

Date	Action	Author
2007-07-08	Authored	de Bono B
2007-07-08	Created	de Bono B
2007-08-06	Reviewed	Trowsdale J
2015-03-27	Authored	Garapati P V
2015-05-13	Reviewed	Barrow AD
2021-05-22	Modified	Shorser S

Entities found in this pathway (10)

Input	UniProt Id	Input	UniProt Id	Input	UniProt Id
B2M	P61769	C3	P01024	CDH1	P12830
COL1A1	P02452	COL1A2	P08123	HLA-B	P01889, P03989, P10319, P18463, P18464, P18465, P30460, P30461, P30462, P30464, P30466, P30475, P30479, P30480, P30481, P30483, P30484, P30485, P30486, P30487, P30488, P30490, P30491, P30492, P30493, P30495, P30498, P30685, Q04826, Q29718, Q29836, Q29940, Q31610, Q31612, Q95365
HLA-C	P04222, P10321, P30499, P30501, P30504, P30505, P30508, P30510, Q07000, Q29865, Q29960, Q29963, Q95604, Q9TNN7	HLA-G	P17693	IGLC1	P0CG04
TSTD1	P78310				

11. Response to elevated platelet cytosolic Ca2+ (R-HSA-76005)

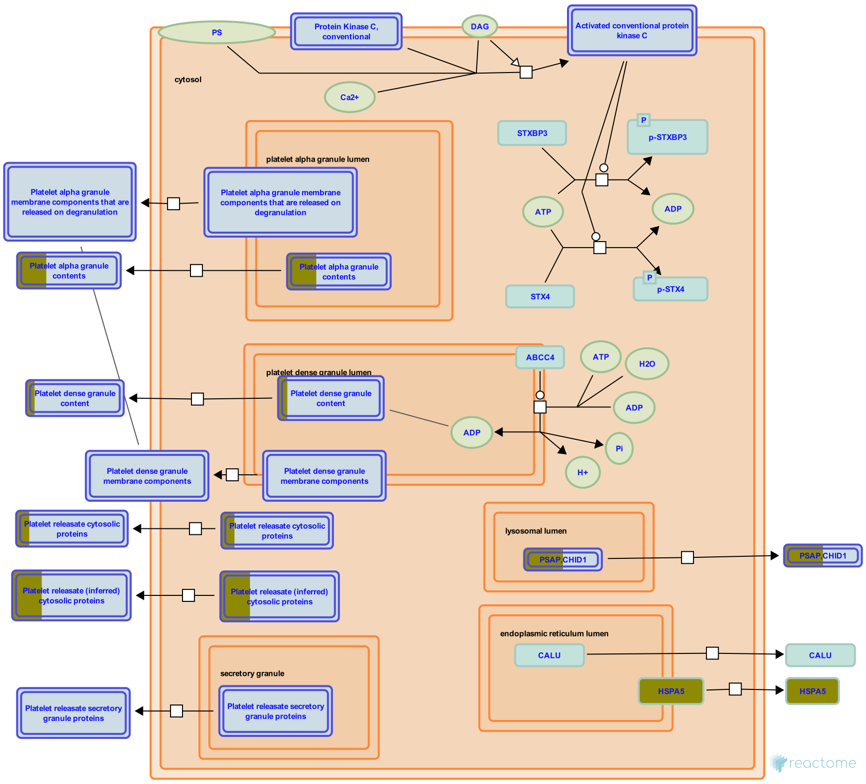

Activation of phospholipase C enzymes results in the generation of second messengers of the phosphatidylinositol pathway. The events resulting from this pathway are a rise in intracellular calcium and activation of Protein Kinase C (PKC). Phospholipase C cleaves the phosphodiester bond in PIP2 to form 1,2 Diacylglycerol (DAG) and 1,4,5-inositol trisphosphate (IP3). IP3 opens Ca2+ channels in the platelet dense tubular system, raising intracellular Ca2+ levels. DAG is a second messenger that regulates a family of Ser/Thr kinases consisting of PKC isozymes (Nishizuka 1995). DAG achieves activation of PKC isozymes by increasing their affinity for phospholipid. Most PKC enzymes are also calcium-dependent, so their activation is in synergy with the rise in intracellular Ca2+. Platelets contain several PKC isoforms that can be activated by DAG and/or Ca2+ (Chang 1997).

References

Walker TR & Watson SP (1993). Synergy between Ca2+ and protein kinase C is the major factor in determining the level of secretion from human platelets. Biochem J, 289, 277-82.

Edit history

Date	Action	Author
2004-08-13	Authored	de Bono B
2004-09-25	Created	Farndale R, Pace NP, de Bono B
2021-05-22	Modified	Shorser S

Entities found in this pathway (23)

Input	UniProt Id	Input	UniProt Id	Input	UniProt Id
A1BG	P04217	A2M	P01023	AHSG	P02765
CAP1	Q01518	CLEC3B	P05452	FGA	P02671

Input	UniProt Id
FGG	P02679
HGF	P14210
LGALS3BP	Q08380
PLG	P00747
SERPINA1	P01009, P01011
VCL	P18206

Input	UniProt Id
FLNA	P21333
HSPA5	P11021
ORM2	P19652
PROS1	P07225
SERPING1	P05155
VEGFA	P15692, P49765

Input	UniProt Id
FN1	P02751
KNG1	P01042
PDGFB	P01127
PSAP	P07602
TGFB1	P01137

12. Adaptive Immune System (R-HSA-1280218)

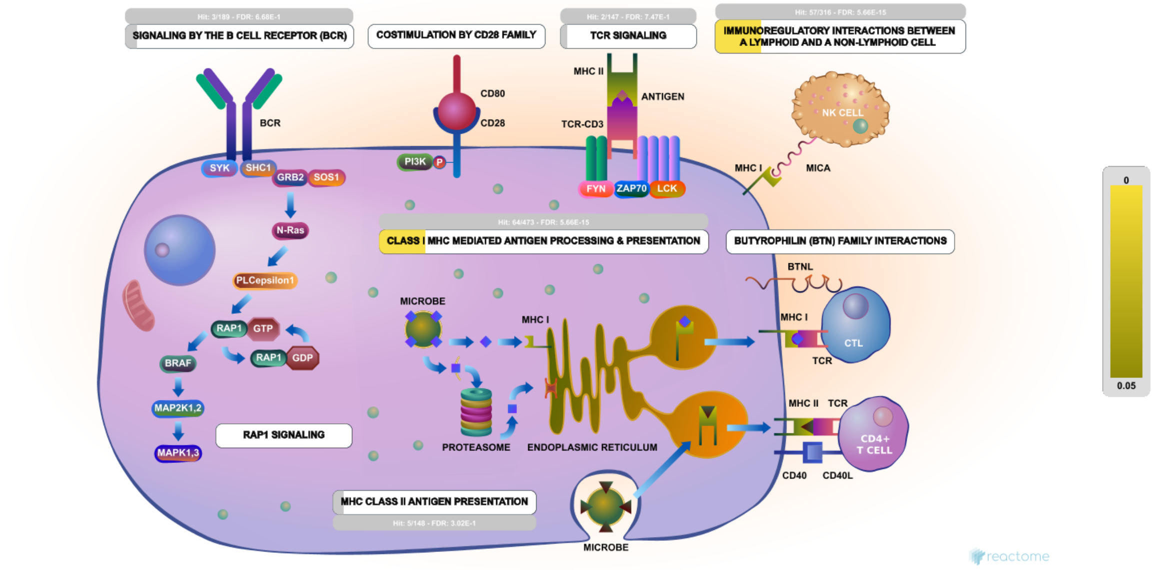

Adaptive immunity refers to antigen-specific immune response efficiently involved in clearing the pathogens. The adaptive immune system is comprised of B and T lymphocytes that express receptors with remarkable diversity tailored to recognize aspects of particular pathogens or antigens. During infection, dendritic cells (DC) which act as sentinels in the peripheral tissues recognize and pick up the pathogen in the form of antigenic determinants and then process these antigens and present them to T cells. These T cells of appropriate specificity respond to the antigen, and either kill the pathogen directly or secrete cytokines that will stimulate B lymphocyte response. B cells provide humoral immunity by secreting antibodies specific for the pathogen or antigen.

References

Minnicozzi M, Sawyer RT & Fenton MJ (2011). Innate immunity in allergic disease. Immunol Rev, 242, 106-27.

Pancer Z & Cooper MD (2006). The evolution of adaptive immunity. Annu Rev Immunol, 24, 497-518.

Janeway CA Jr & Medzhitov R (2002). Innate immune recognition. Annu Rev Immunol, 20, 197-216.

Edit history

Date	Action	Author
2011-05-12	Created	Garapati P V
2011-05-22	Edited	May B, Jupe S, Garapati P V, de Bono B
2011-05-22	Authored	May B, Jupe S, Garapati P V, de Bono B
2011-05-28	Reviewed	Heemskerk JW, Bluestone JA, Elliott T, Trowsdale J, Esensten J
2021-05-22	Modified	Shorser S

Entities found in this pathway (23)

Input	UniProt Id	Input	UniProt Id	Input	UniProt Id
ARF1	P84077	B2M	P28068, P61769	C3	P01024
CALR	P27797	CAP1	P47755	CDH1	P12830, Q9UM11
CLTC	Q00610, Q00610-1	COL1A1	P02452	COL1A2	P08123
FGA	P02671	FGG	P02679	HLA-B	P01889, P03989, P10319, P18463, P18464, P18465, P30460, P30461, P30462, P30464, P30466, P30475, P30479, P30480, P30481, P30483, P30484, P30485, P30486, P30487, P30488, P30490, P30491, P30492, P30493, P30495, P30498, P30685, Q04826, Q29718, Q29836, Q29940, Q31610, Q31612, Q95365
HLA-C	P04222, P10321, P30499, P30501, P30504, P30505, P30508, P30510, Q07000, Q29865, Q29960, Q29963, Q95604, Q9TNN7	HLA-G	P17693	HMGB1	P09429
HSPA5	P11021	HUWE1	Q7Z6Z7, Q8IYU2	IGLC1	P0CG04
PDIA3	P30101	PSMA7	O14818, Q8TAA3	S100A9	P06702
TSTD1	P78310	UBA3	Q8TBC4		

13. Immune System (R-HSA-168256)

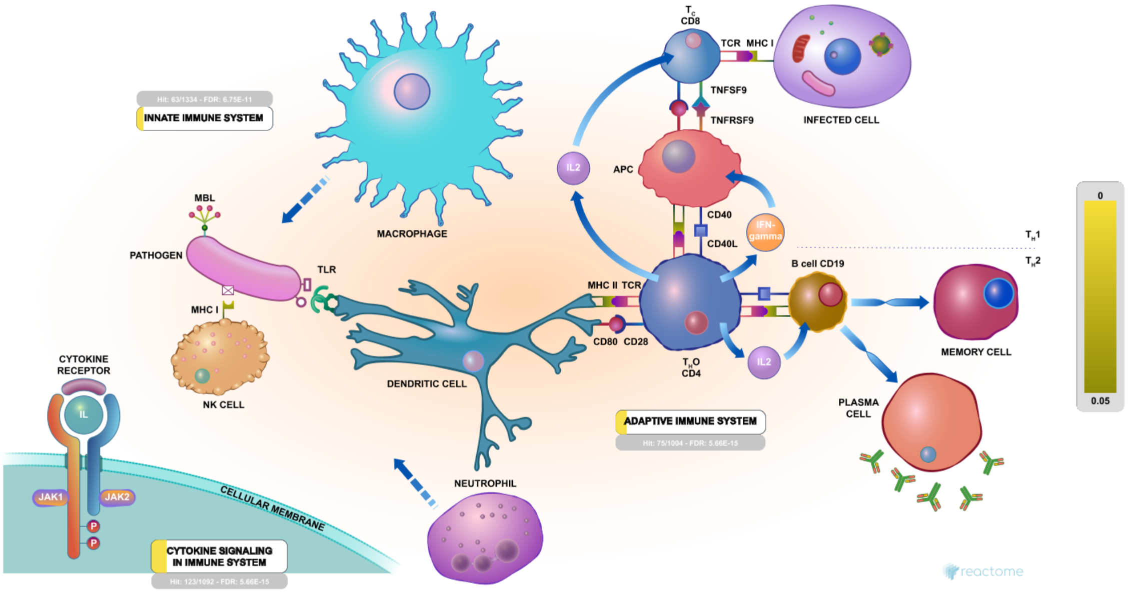

Humans are exposed to millions of potential pathogens daily, through contact, ingestion, and inhalation. Our ability to avoid infection depends on the adaptive immune system and during the first critical hours and days of exposure to a new pathogen, our innate immune system.

References

Edit history

Date	Action	Author
2005-11-12	Created	Gillespie ME
2006-03-30	Authored	Luo F, Ouwehand WH, Gillespie ME, de Bono B
2006-04-19	Reviewed	Zwaginga JJ, D'Eustachio P, Gay NJ, Gale M Jr
2021-05-22	Modified	Shorser S

Entities found in this pathway (85)

Input	UniProt Id	Input	UniProt Id	Input	UniProt Id
A1BG	P04217	ACTR3	P61158	AGL	P35573
AHSG	P02765	ANXA1	P04083	ANXA2	P07355
APOB	P04114	ARF1	P84077	B2M	P28068, P61769
C1QA	P02745	C1QB	P02746, P02747	C2	P06681
C3	P01024	C4A	P0C0L4, P0C0L5	C4B	P0C0L4, P0C0L5
C4BPA	P04003, P15529	C5	P01031	CALR	P27797
CAP1	P47755, Q01518, Q13114	CCL2	P13500	CCL3L3	P16619
CCL4	O00626, P13236, P51671	CCL5	P13501	CCT2	P78371
CDH1	P12830, Q9UM11	CFB	P00751	CLTC	Q00610, Q00610-1
COL1A1	P02452	COL1A2	P08123	COTL1	Q14019

Input	UniProt Id	Input	UniProt Id	Input	UniProt Id
CSF2	P04141	CSF3	P09919	CXCL1	P09341
CXCL2	P19875	CXCL8	P09341, P10145	EEF2	P13639
F2	P00734	FGA	P02671	FGG	P02679
FLNA	P21333	FN1	P02751	GPI	P06744
GSTP1	P09211	HGF	P14210	HLA-B	P01889, P03989, P10319, P18463, P18464, P18465, P30460, P30461, P30462, P30464, P30466, P30475, P30479, P30480, P30481, P30483, P30484, P30485, P30486, P30487, P30488, P30490, P30491, P30492, P30493, P30495, P30498, P30685, Q04826, Q29718, Q29836, Q29940, Q31610, Q31612, Q95365
HLA-C	P04222, P10321, P30499, P30501, P30504, P30505, P30508, P30510, Q07000, Q29865, Q29960, Q29963, Q95604, Q9TNN7	HLA-G	P17693	HMGB1	P09429
HNRNPA2B1	P22626	HP	P00738	HSP90AA1	P07900
HSP90B1	P14625	HSPA5	P11021	HUWE1	Q7Z6Z7, Q8IYU2
IFNG	P01579	IGLC1	P0CG04	IL10	P22301
IL1B	P01583, P01584	IL3	P08700	IL4	P05112
IL6	P05231	IL7	P13232	LCN2	P80188
LTA	P01374	LTF	P02788	MAP2K6	P52564
ORM2	P19652	OSM	P13725	PDIA3	P30101
PROS1	P07225	PSAP	P07602, Q8IWL1, Q8IWL2	PSMA7	O14818, Q8TAA3
S100A11	P31949	S100A9	P06702	SDC1	P18827
SERPINA1	P01009, P01011	SERPING1	P05155	TGFB1	P01137
TNF	P01375	TSTD1	P78310	UBA3	Q8TBC4
VAT1	Q99536	VCL	P18206	VEGFA	P15692
VTN	P04004				

Input	Ensembl Id	Input	Ensembl Id	Input	Ensembl Id
ANXA1	ENSG00000135046	ANXA2	ENSG00000182718	ARF1	ENSG00000143761
B2M	ENSG00000166710	CCL2	ENSG00000108691	CCL3L3	ENSG00000277768
CCL4	ENSG00000275302	CCL5	ENSG00000271503	COL1A2	ENSG00000164692
CSF2	ENSG00000164400	CSF3	ENSG00000108342	CXCL1	ENSG00000163739
CXCL2	ENSG00000081041	CXCL8	ENSG00000169429	FN1	ENSG00000115414
HGF	ENSG00000019991	HLA-B	ENSG00000234745	HLA-C	ENSG00000204525
HLA-G	ENSG00000204632	HNRNPA2B1	ENSG00000122566	HSP90AA1	ENSG00000080824
IFNG	ENSG00000111537	IL10	ENSG00000136634	IL1B	ENSG00000125538, ENST00000263341
IL6	ENSG00000136244	LCN2	ENSG00000148346	OSM	ENSG00000099985
TGFB1	ENSG00000105329	TNF	ENSG00000232810	VEGFA	ENSG00000112715

14. Signaling by Interleukins (R-HSA-449147)

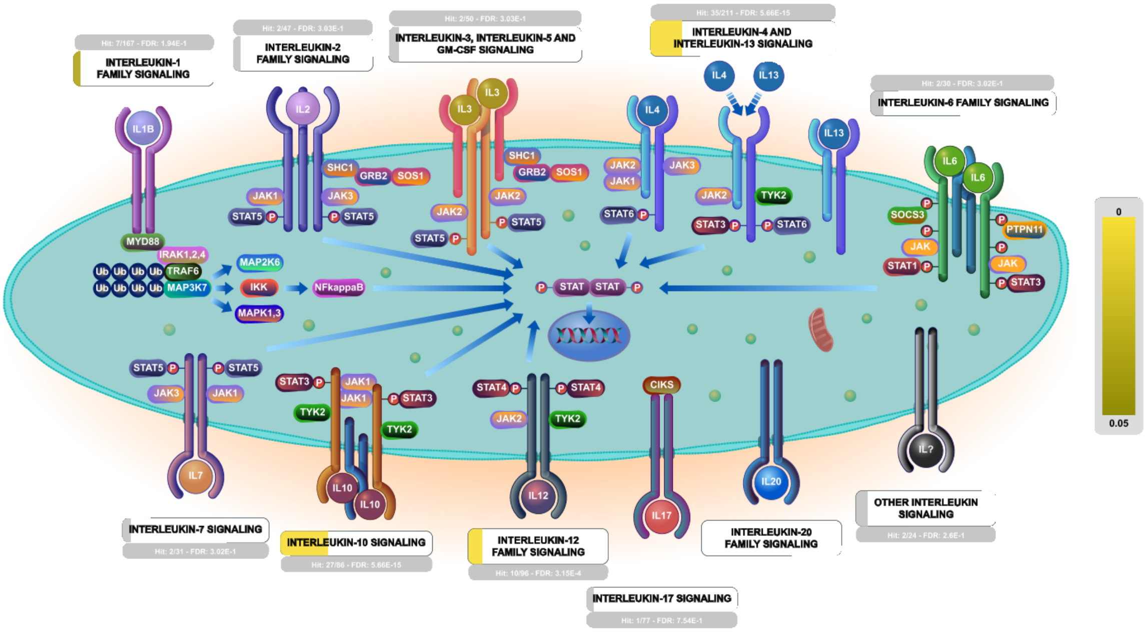

Cellular compartments: plasma membrane.

Interleukins are low molecular weight proteins that bind to cell surface receptors and act in an autocrine and/or paracrine fashion. They were first identified as factors produced by leukocytes but are now known to be produced by many other cells throughout the body. They have pleiotropic effects on cells which bind them, impacting processes such as tissue growth and repair, hematopoietic homeostasis, and multiple levels of the host defense against pathogens where they are an essential part of the immune system.

References

Vosshenrich CA & Di Santo JP (2002). Interleukin signaling. Curr Biol, 12, R760-3.

Dinarello CA (2009). Immunological and inflammatory functions of the interleukin-1 family. Annu Rev Immunol, 27, 519-50.

Akdis M, Aab A, Altunbulakli C, Azkur K, Costa RA, Crameri R, ... Akdis CA (2016). Interleukins (from IL-1 to IL-38), interferons, transforming growth factor , and TNF-: Receptors, functions, and roles in diseases. J. Allergy Clin. Immunol., 138, 984-1010.

Edit history

Date	Action	Author
2009-11-27	Created	Jupe S
2010-05-17	Reviewed	Pinteaux E
2010-05-17	Authored	Ray KP
2010-05-26	Edited	Jupe S
2021-05-22	Modified	Shorser S

Entities found in this pathway (34)

Input	UniProt Id	Input	UniProt Id	Input	UniProt Id
ANXA1	P04083	ANXA2	P07355	ARF1	P84077
CCL2	P13500	CCL3L3	P16619	CCL4	O00626, P13236, P51671
CCL5	P13501	COL1A2	P08123	CSF2	P04141
CSF3	P09919	CXCL1	P09341	CXCL2	P19875
CXCL8	P09341, P10145	FN1	P02751	HGF	P14210
HMGB1	P09429	HNRNPA2B1	P22626	HSP90AA1	P07900
HSP90B1	P14625	IFNG	P01579	IL10	P22301
IL1B	P01583, P01584	IL3	P08700	IL4	P05112
IL6	P05231	IL7	P13232	LCN2	P80188
MAP2K6	P52564	OSM	P13725	PSMA7	O14818, Q8TAA3
SDC1	P18827	TGFB1	P01137	TNF	P01375
VEGFA	P15692				

Input	Ensembl Id	Input	Ensembl Id	Input	Ensembl Id
ANXA1	ENSG00000135046	ANXA2	ENSG00000182718	ARF1	ENSG00000143761
CCL2	ENSG00000108691	CCL3L3	ENSG00000277768	CCL4	ENSG00000275302
CCL5	ENSG00000271503	COL1A2	ENSG00000164692	CSF2	ENSG00000164400
CSF3	ENSG00000108342	CXCL1	ENSG00000163739	CXCL2	ENSG00000081041
CXCL8	ENSG00000169429	FN1	ENSG00000115414	HGF	ENSG00000019991
HNRNPA2B1	ENSG00000122566	HSP90AA1	ENSG00000080824	IFNG	ENSG00000111537
IL10	ENSG00000136634	IL1B	ENSG00000125538	IL6	ENSG00000136244
LCN2	ENSG00000148346	OSM	ENSG00000099985	TGFB1	ENSG00000105329
TNF	ENSG00000232810	VEGFA	ENSG00000112715		

15. Cytokine Signaling in Immune system (R-HSA-1280215)

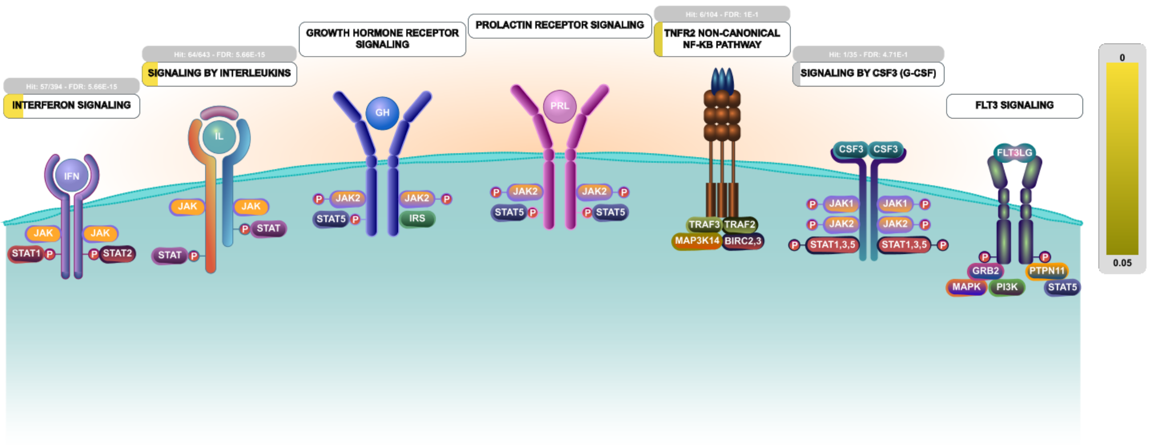

Cytokines are small proteins that regulate and mediate immunity, inflammation, and hematopoiesis. They are secreted in response to immune stimuli, and usually act briefly, locally, at very low concentrations. Cytokines bind to specific membrane receptors, which then signal the cell via second messengers, to regulate cellular activity.

References

Oppenheim J & Feldmann M (2002). *Cytokines and the immune system, Cytokine Reference* .

IMMPORT:Bioinformatics for the future of immunology. Retrieved from https://www.immport.org/immportWeb/queryref/geneListSummary.do

COPE. Retrieved from http://www.copewithcytokines.org/cope.cgi

Santamaria P (2003). Cytokines and chemokines in autoimmune disease: an overview. Adv Exp Med Biol, 520, 1-7.

Edit history

Date	Action	Author
2011-05-12	Created	Garapati P V
2011-05-22	Edited	Ray KP, Jupe S, Garapati P V
2011-05-22	Authored	Ray KP, Jupe S, Garapati P V
2011-05-29	Reviewed	Abdul-Sater AA, Schindler C, Pinteaux E
2021-05-22	Modified	Shorser S

Entities found in this pathway (42)

Input	UniProt Id	Input	UniProt Id	Input	UniProt Id
ANXA1	P04083	ANXA2	P07355	ARF1	P84077
B2M	P61769	CAP1	Q13114	CCL2	P13500

Input	UniProt Id	Input	UniProt Id	Input	UniProt Id
CCL3L3	P16619	CCL4	O00626, P13236, P51671	CCL5	P13501
COL1A2	P08123	CSF2	P04141	CSF3	P09919
CXCL1	P09341	CXCL2	P19875	CXCL8	P09341, P10145
FLNA	P21333	FN1	P02751	HGF	P14210
HLA-B	P01889, P03989, P10319, P18463, P18464, P18465, P30460, P30461, P30462, P30464, P30466, P30475, P30479, P30480, P30481, P30483, P30484, P30485, P30486, P30487, P30488, P30490, P30491, P30492, P30493, P30495, P30498, P30685, Q04826, Q29718, Q29836, Q29940, Q31610, Q31612, Q95365	HLA-C	P04222, P10321, P30499, P30501, P30504, P30505, P30508, P30510, Q07000, Q29865, Q29960, Q29963, Q95604, Q9TNN7	HLA-G	P17693
HMGB1	P09429	HNRNPA2B1	P22626	HSP90AA1	P07900
HSP90B1	P14625	IFNG	P01579	IL10	P22301
IL1B	P01583, P01584	IL3	P08700	IL4	P05112
IL6	P05231	IL7	P13232	LCN2	P80188
LTA	P01374	MAP2K6	P52564	OSM	P13725
PSMA7	O14818, Q8TAA3	SDC1	P18827	TGFB1	P01137
TNF	P01375	UBA3	Q8TBC4	VEGFA	P15692

Input	Ensembl Id	Input	Ensembl Id	Input	Ensembl Id
ANXA1	ENSG00000135046	ANXA2	ENSG00000182718	ARF1	ENSG00000143761
B2M	ENSG00000166710	CCL2	ENSG00000108691	CCL3L3	ENSG00000277768
CCL4	ENSG00000275302	CCL5	ENSG00000271503	COL1A2	ENSG00000164692
CSF2	ENSG00000164400	CSF3	ENSG00000108342	CXCL1	ENSG00000163739
CXCL2	ENSG00000081041	CXCL8	ENSG00000169429	FN1	ENSG00000115414
HGF	ENSG00000019991	HLA-B	ENSG00000234745	HLA-C	ENSG00000204525
HLA-G	ENSG00000204632	HNRNPA2B1	ENSG00000122566	HSP90AA1	ENSG00000080824
IFNG	ENSG00000111537	IL10	ENSG00000136634	IL1B	ENSG00000125538
IL6	ENSG00000136244	LCN2	ENSG00000148346	OSM	ENSG00000099985
TGFB1	ENSG00000105329	TNF	ENSG00000232810	VEGFA	ENSG00000112715

16. Interferon Signaling (R-HSA-913531)

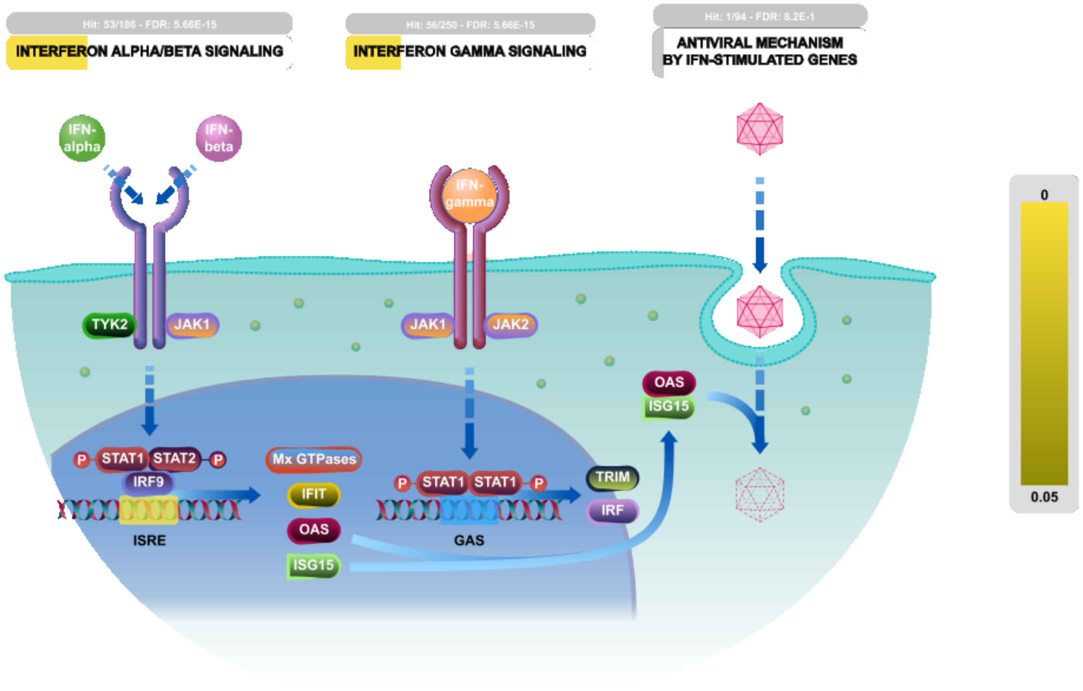

Interferons (IFNs) are cytokines that play a central role in initiating immune responses, especially antiviral and antitumor effects. There are three types of IFNs:Type I (IFN-alpha, -beta and others, such as omega, epsilon, and kappa), Type II (IFN-gamma) and Type III (IFN-lamda). In this module we are mainly focusing on type I IFNs alpha and beta and type II IFN-gamma. Both type I and type II IFNs exert their actions through cognate receptor complexes, IFNAR and IFNGR respectively, present on cell surface membranes. Type I IFNs are broadly expressed heterodimeric receptors composed of the IFNAR1 and IFNAR2 subunits, while the type II IFN receptor consists of IFNGR1 and IFNGR2. Type III interferon lambda has three members: lamda1 (IL-29), lambda2 (IL-28A), and lambda3 (IL-28B) respectively. IFN-lambda signaling is initiated through unique heterodimeric receptor composed of IFN-LR1/IF-28Ralpha and IL10R2 chains.

Type I IFNs typically recruit JAK1 and TYK2 proteins to transduce their signals to STAT1 and 2; in combination with IRF9 (IFN-regulatory factor 9), these proteins form the heterotrimeric complex ISGF3. In nucleus ISGF3 binds to IFN-stimulated response elements (ISRE) to promote gene induction.

Type II IFNs in turn rely upon the activation of JAKs 1 and 2 and STAT1. Once activated, STAT1 dimerizes to form the transcriptional regulator GAF (IFNG activated factor) and this binds to the IFNG activated sequence (GAS) elements and initiate the transcription of IFNG-responsive genes.

Like type I IFNs, IFN-lambda recruits TYK2 and JAK1 kinases and then promote the phosphorylation of STAT1/2, and induce the ISRE3 complex formation.

References

Platanias LC (2005). Mechanisms of type-I- and type-II-interferon-mediated signalling. Nat Rev Immunol, 5, 375-86.

Gough DJ, Levy DE, Johnstone RW & Clarke CJ (2008). IFNgamma signaling-does it mean JAK-STAT?. Cytokine Growth Factor Rev, 19, 383-94.

Schroder K, Hertzog PJ, Ravasi T & Hume DA (2004). Interferon-gamma: an overview of signals, mechanisms and functions. J Leukoc Biol, 75, 163-89.

Bonjardim CA, Ferreira PC & Kroon EG (2009). Interferons: signaling, antiviral and viral evasion. Immunol Lett, 122, 1-11.

Uddin S & Platanias LC (2004). Mechanisms of type-I interferon signal transduction. J Biochem Mol Biol, 37, 635-41.

Edit history

Date	Action	Author
2010-07-07	Edited	Garapati P V
2010-07-07	Authored	Garapati P V
2010-07-16	Created	Garapati P V
2010-08-17	Reviewed	Abdul-Sater AA, Schindler C
2021-05-22	Modified	Shorser S

Entities found in this pathway (6)

Input	UniProt Id	Input	UniProt Id	Input	UniProt Id
B2M	P61769	FLNA	P21333	HLA-B	P01889, P03989, P10319, P18463, P18464, P18465, P30460, P30461, P30462, P30464, P30466, P30475, P30479, P30480, P30481, P30483, P30484, P30485, P30486, P30487, P30488, P30490, P30491, P30492, P30493, P30495, P30498, P30685, Q04826, Q29718, Q29836, Q29940, Q31610, Q31612, Q95365
HLA-C	P04222, P10321, P30499, P30501, P30504, P30505, P30508, P30510, Q07000, Q29865, Q29960, Q29963, Q95604, Q9TNN7	HLA-G	P17693	IFNG	P01579

Input	Ensembl Id	Input	Ensembl Id
B2M	ENSG00000166710	HLA-B	ENSG00000234745
HLA-C	ENSG00000204525	HLA-G	ENSG00000204632

17. Interferon alpha/beta signaling (R-HSA-909733)

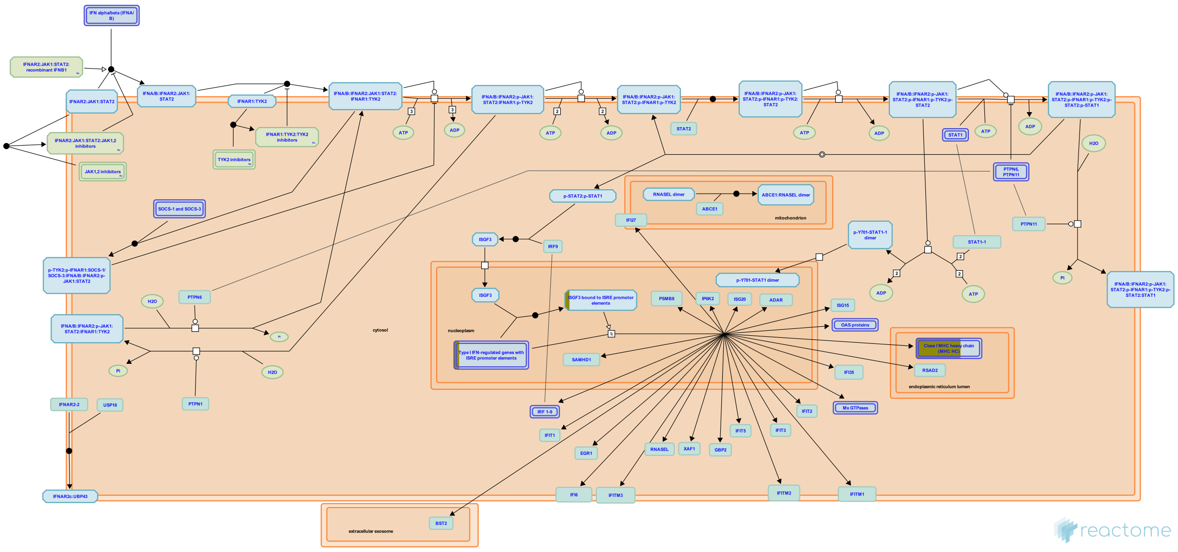

Type I interferons (IFNs) are composed of various genes including IFN alpha (IFNA), beta (IFNB), omega, epsilon, and kappa. In humans the IFNA genes are composed of more than 13 subfamily genes, whereas there is only one IFNB gene. The large family of IFNA/B proteins all bind to a single receptor which is composed of two distinct chains: IFNAR1 and IFNAR2. The IFNA/B stimulation of the IFNA receptor complex leads to the formation of two transcriptional activator complexes: IFNA-activated-factor (AAF), which is a homodimer of STAT1 and IFN-stimulated gene factor 3 (ISGF3), which comprises STAT1, STAT2 and a member of the IRF family, IRF9/P48. AAF mediates activation of the IRF-1 gene by binding to GAS (IFNG-activated site), whereas ISGF3 activates several IFN-inducible genes including IRF3 and IRF7.

References

Uzé G, Schreiber G, Piehler J & Pellegrini S (2007). The receptor of the type I interferon family. Curr Top Microbiol Immunol, 316, 71-95.

Gauzzi MC, Velazquez L, McKendry R, Mogensen KE, Fellous M & Pellegrini S (1996). Interferon-alpha-dependent activation of Tyk2 requires phosphorylation of positive regulatory tyrosines by another kinase. J Biol Chem, 271, 20494-500.

Yan H, Krishnan K, Greenlund AC, Gupta S, Lim JT, Schreiber RD, ... Krolewski JJ (1996). Phosphorylated interferon-alpha receptor 1 subunit (IFNaR1) acts as a docking site for the latent form of the 113 kDa STAT2 protein. EMBO J, 15, 1064-74.

Li X, Leung S, Qureshi S, Darnell JE Jr & Stark GR (1996). Formation of STAT1-STAT2 heterodimers and their role in the activation of IRF-1 gene transcription by interferon-alpha. J Biol Chem, 271, 5790-4.

Edit history

Date	Action	Author
2010-07-07	Edited	Garapati P V
2010-07-07	Authored	Garapati P V
2010-07-07	Created	Garapati P V
2010-08-17	Reviewed	Abdul-Sater AA, Schindler C

Date	Action	Author
2021-05-22	Modified	Shorser S

Entities found in this pathway (3)

Input	UniProt Id	Input	UniProt Id	Input	UniProt Id
HLA-B	P01889, P03989, P10319, P18463, P18464, P18465, P30460, P30461, P30462, P30464, P30466, P30475, P30479, P30480, P30481, P30483, P30484, P30485, P30486, P30487, P30488, P30490, P30491, P30492, P30493, P30495, P30498, P30685, Q04826, Q29718, Q29836, Q29940, Q31610, Q31612, Q95365	HLA-C	P04222, P10321, P30499, P30501, P30504, P30505, P30508, P30510, Q07000, Q29865, Q29960, Q29963, Q95604, Q9TNN7	HLA-G	P17693

Input	Ensembl Id	Input	Ensembl Id	Input	Ensembl Id
HLA-B	ENSG00000234745	HLA-C	ENSG00000204525	HLA-G	ENSG00000204632

18. Platelet activation, signaling and aggregation (R-HSA-76002)

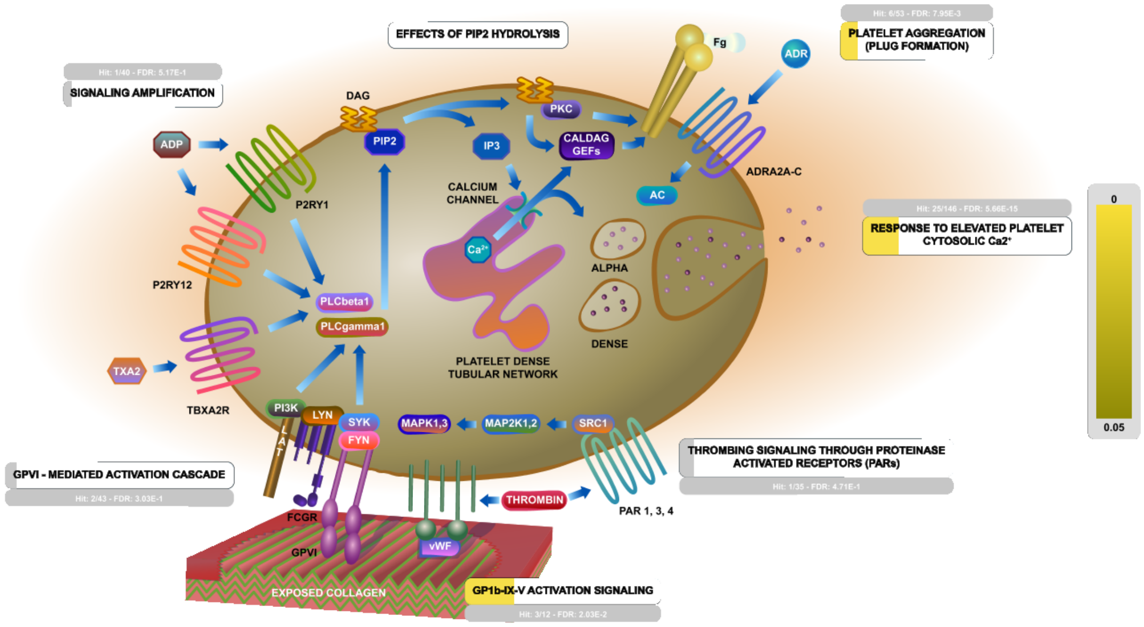

Platelet activation begins with the initial binding of adhesive ligands and of the excitatory platelet agonists (released or generated at the sites of vascular trauma) to cognate receptors on the platelet membrane (Ruggeri 2002). Intracellular signaling reactions then enhance the adhesive and procoagulant properties of tethered platelets or of platelets circulating in the proximity. Once platelets have adhered they degranulate, releasing stored secondary agents such as ADP, ATP, and synthesize thromboxane A2. These amplify the response, activating and recruiting further platelets to the area and promoting platelet aggregation. These amplify the response, activating and recruiting further platelets to the area and promoting platelet aggregation. Adenosine nucleotides signal through P2 purinergic receptors on the platelet membrane. ADP activates P2Y1 and P2Y12, which signal via both the alpha and gamma:beta components of the heterotrimeric G-protein (Hirsch et al. 2001, 2006),
while ATP activates the ionotropic P2X1 receptor (Kunapuli et al. 2003). Activation of these receptors initiates a complex signaling cascade that ultimately results in platelet activation, aggregation and thrombus formation (Kahner et al. 2006).

Integrin AlphaIIbBeta3 is the most abundant platelet receptor, with 40 000 to 80 000 copies per resting platelet, acting as a major receptor for fibrinogen and other adhesive molecules (Wagner et al. 1996). Activation of AlphaIIbBeta3 enhances adhesion and leads to platelet-platelet interactions, and thus aggregation (Philips et al. 1991). GP VI is the most potent collagen receptor initiating signal generation, an ability derived from its interaction with the FcRI gamma chain. This results in the phosphorylation of the gamma-chain by non-receptor tyrosine kinases of the Src family (1). The phosphotyrosine motif is recognized by the SH2 domains of Syk, a tyrosine kinase. This association activates the Syk enzyme, leading to activation (by tyrosine phosphorylation) of PLC gamma2 (2). Thrombin is an important platelet agonist generated on the membrane of stimulated platelets. Thrombin acts via cell surface Protease Activated Receptors (PARs). PARs are G-protein coupled receptors activated by a proteolytic cleavage in an extracellular loop (Vu, 1991) (3). Activated PARs signal via G alpha q (4) and via the beta:gamma component of the G-protein (5). Both stimulate PLC giving rise to PIP2 hydrolysis and consequent activation of PI3K (6). PLCgamma2 activation also gives rise to IP3 (7) which stimulates the IP3 receptor (8) leading to increased intracellular calcium. Platelet activation further results in the scramblase-mediated transport of negatively-charged phospholipids to the platelet surface. These phospholipids provide a catalytic surface (with the charge provided by phosphatidylserine and phosphatidylethanolamine) for the tenase complex (formed by the activated forms of the blood coagulation factors factor VIII and factor I).

References

Wagner CL, Mascelli MA, Neblock DS, Weisman HF, Coller BS & Jordan RE (1996). Analysis of GPIIb/IIIa receptor number by quantification of 7E3 binding to human platelets. Blood, 88, 907-14.

Phillips DR, Charo IF & Scarborough RM (1991). GPIIb-IIIa: the responsive integrin. Cell, 65, 359-62.

Vu TK, Hung DT, Wheaton VI & Coughlin SR (1991). Molecular cloning of a functional thrombin receptor reveals a novel proteolytic mechanism of receptor activation. Cell, 64, 1057-68.

Kahner BN, Shankar H, Murugappan S, Prasad GL & Kunapuli SP (2006). Nucleotide receptor signaling in platelets. J Thromb Haemost, 4, 2317-26.

Kunapuli SP, Dorsam RT, Kim S & Quinton TM (2003). Platelet purinergic receptors. Curr Opin Pharmacol, 3, 175-80.

Edit history

Date	Action	Author
2004-08-13	Authored	de Bono B
2004-09-25	Created	Farndale R, Pace NP, de Bono B
2010-06-07	Revised	Jupe S
2010-06-07	Reviewed	Kunapuli SP
2021-05-22	Modified	Shorser S

Entities found in this pathway (27)

Input	UniProt Id	Input	UniProt Id	Input	UniProt Id
A1BG	P04217	A2M	P01023	AHSG	P02765
CAP1	Q01518	CLEC3B	P05452	COL1A1	P02452
COL1A2	P08123	F2	P00734	FGA	P02671
FGG	P02679	FLNA	P21333	FN1	P02751
GNAI2	P04899	HGF	P14210	HSPA5	P11021
KNG1	P01042	LGALS3BP	Q08380	ORM2	P19652
PDGFB	P01127	PLG	P00747	PROS1	P07225
PSAP	P07602	SERPINA1	P01009, P01011	SERPING1	P05155
TGFB1	P01137	VCL	P18206	VEGFA	P15692, P49765

19. Innate Immune System (R-HSA-168249)

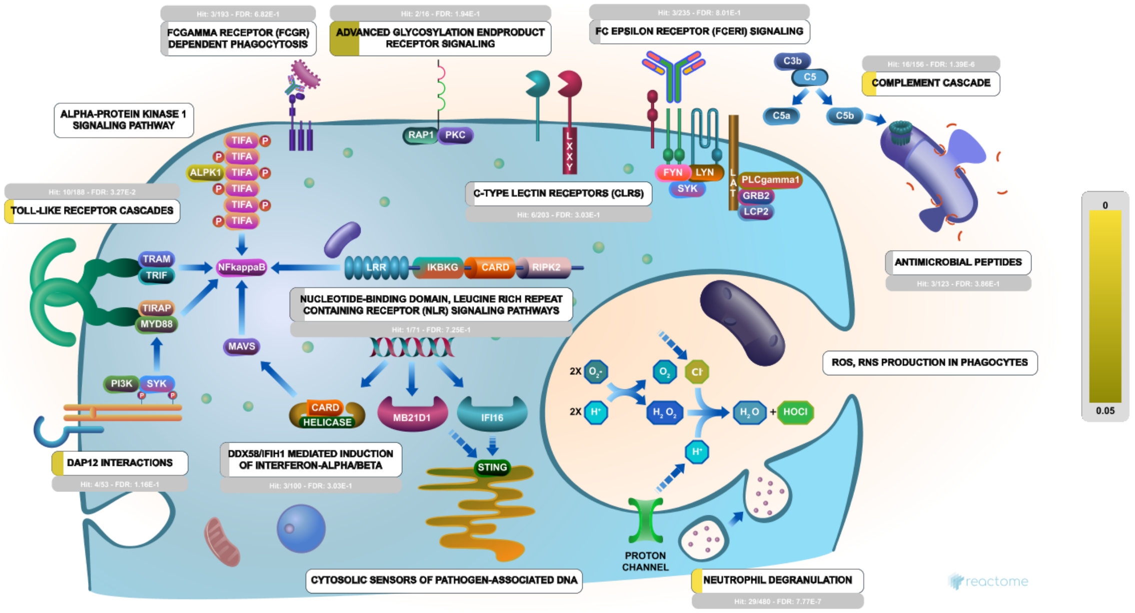

Innate immunity encompases the nonspecific part of immunity tha are part of an individual's natural biologic makeup

References

Edit history

Date	Action	Author
2005-11-12	Created	Gillespie ME
2021-05-22	Modified	Shorser S

Entities found in this pathway (51)

Input	UniProt Id	Input	UniProt Id	Input	UniProt Id
A1BG	P04217	ACTR3	P61158	AGL	P35573
AHSG	P02765	ANXA2	P07355	APOB	P04114
B2M	P61769	C1QA	P02745	C1QB	P02746, P02747
C2	P06681	C3	P01024	C4A	P0C0L4, P0C0L5
C4B	P0C0L4, P0C0L5	C4BPA	P04003, P15529	C5	P01031
CAP1	P47755, Q01518, Q13114	CCT2	P78371	CFB	P00751
COTL1	Q14019	CXCL1	P09341	CXCL8	P09341
EEF2	P13639	F2	P00734	FGA	P02671
FGG	P02679	GPI	P06744	GSTP1	P09211
HLA-B	P01889, Q95365	HLA-C	P04222, P10321, P30504	HMGB1	P09429
HP	P00738	HSP90AA1	P07900	HSP90B1	P14625
HUWE1	Q7Z6Z7	IGLC1	P0CG04	IL1B	P01584
LCN2	P80188	LTF	P02788	MAP2K6	P52564
ORM2	P19652	PROS1	P07225	PSAP	P07602, Q8IWL1, Q8IWL2
PSMA7	O14818, Q8TAA3	S100A11	P31949	S100A9	P06702
SERPINA1	P01009, P01011	SERPING1	P05155	UBA3	Q8TBC4
VAT1	Q99536	VCL	P18206	VTN	P04004

Input	Ensembl Id
IL1B	ENSG00000125538, ENST00000263341

20. Regulation of Insulin-like Growth Factor (IGF) transport and uptake by Insulin-like Growth Factor Binding Proteins (IGFBPs) (R-HSA-381426)

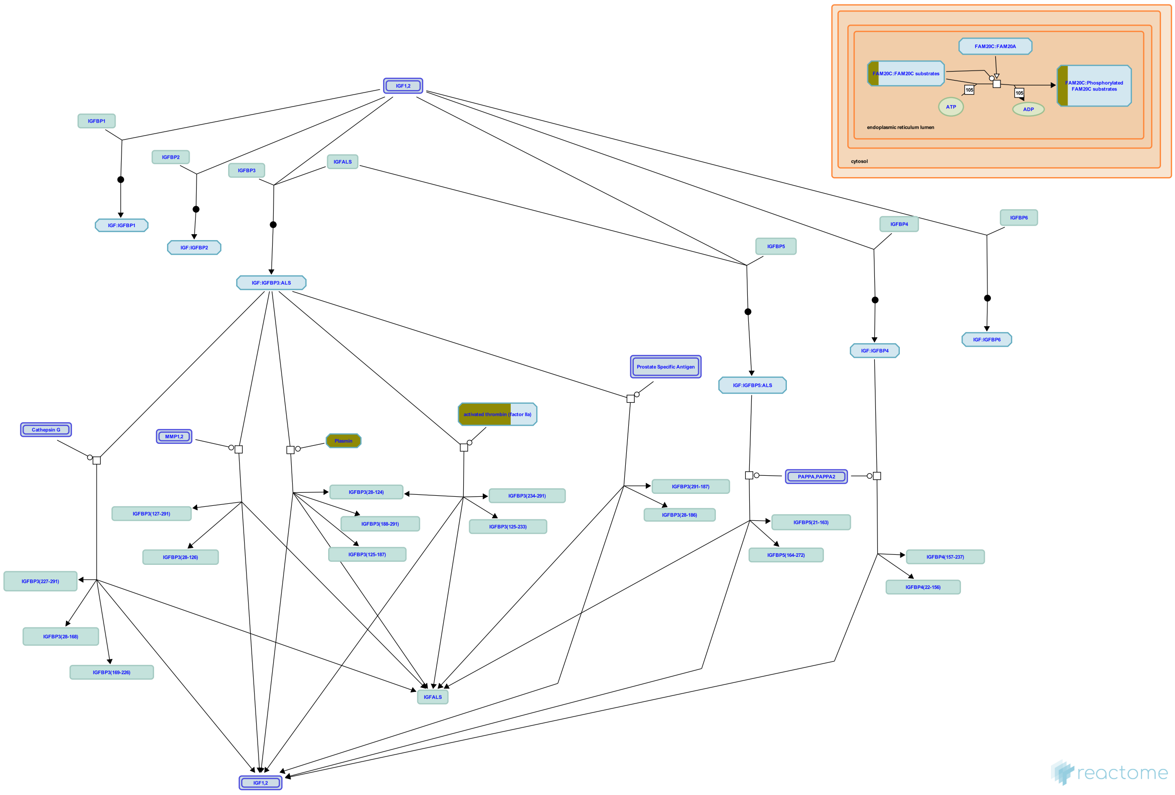

Cellular compartments: extracellular region.

The family of Insulin like Growth Factor Binding Proteins (IGFBPs) share 50% amino acid identity with conserved N terminal and C terminal regions responsible for binding Insulin like Growth Factors I and II (IGF I and IGF II). Most circulating IGFs are in complexes with IGFBPs, which are believed to increase the residence of IGFs in the body, modulate availability of IGFs to target receptors for IGFs, reduce insulin like effects of IGFs, and act as signaling molecules independently of IGFs.
About 75% of circulating IGFs are in 1500 220 KDa complexes with IGFBP3 and ALS. Such complexes are too large to pass the endothelial barrier. The remaining 20 25% of IGFs are bound to other IGFBPs in 40 50 KDa complexes. IGFs are released from IGF:IGFBP complexes by proteolysis of the IGFBP. IGFs become active after release, however IGFs may also have activity when still bound to some IGFBPs.
IGFBP1 is enriched in amniotic fluid and is produced in the liver under control of insulin (insulin suppresses production). IGFBP1 binding stimulates IGF function. It is unknown which if any protease degrades IGFBP1.
IGFBP2 is enriched in cerebrospinal fluid; its binding inhibits IGF function. IGFBP2 is not significantly degraded in circulation.
IGFB3, which binds most IGF in the body is enriched in follicular fluid and found in many other tissues. IGFBP 3 may be cleaved by plasmin, thrombin, Prostate specific Antigen (PSA, KLK3), Matrix Metalloprotease-1 (MMP1), and Matrix Metalloprotease-2 (MMP2). IGFBP3 also binds extracellular matrix and binding lowers its affinity for IGFs. IGFBP3 binding stimulates the effects of IGFs.
IGFBP4 acts to inhibit IGF function and is cleaved by Pregnancy associated Plasma Protein A (PAPPA) to release IGF.
IGFBP5 is enriched in bone matrix; its binding stimulates IGF function. IGFBP5 is cleaved by Pregnancy Associated Plasma Protein A2 (PAPPA2), ADAM9, complement C1s from smooth muscle, and thrombin. Only the cleavage site for PAPPA2 is known.
IGFBP6 is enriched in cerebrospinal fluid. It is unknown which if any protease degrades IGFBP6.

References

Firth SM & Baxter RC (2002). Cellular actions of the insulin-like growth factor binding proteins. Endocr Rev, 23, 824-54.

Hoeflich A, Reisinger R, Lahm H, Kiess W, Blum WF, Kolb HJ, ... Wolf E (2001). Insulin-like growth factor-binding protein 2 in tumorigenesis: protector or promoter?. Cancer Res, 61, 8601-10.

Mohan S & Baylink DJ (2002). IGF-binding proteins are multifunctional and act via IGF-dependent and -independent mechanisms. J Endocrinol, 175, 19-31.

Holly J & Perks C (2006). The role of insulin-like growth factor binding proteins. Neuroendocrinology, 83, 154-60.

Zhou R, Diehl D, Hoeflich A, Lahm H & Wolf E (2003). IGF-binding protein-4: biochemical characteristics and functional consequences. J Endocrinol, 178, 177-93.

Edit history

Date	Action	Author
2008-11-20	Edited	May B, Gopinathrao G
2008-11-20	Created	May B
2008-12-02	Reviewed	Matthews L, D'Eustachio P, Gillespie ME

Date	Action	Author
2021-05-22	Modified	Shorser S

Entities found in this pathway (18)

Input	UniProt Id	Input	UniProt Id	Input	UniProt Id
AHSG	P02765	APOB	P04114	APOE	P02649
C3	P01024	C4A	P0C0L4	C4B	P0C0L4
CP	P00450	F2	P00734	FGA	P02671
FGG	P02679	FN1	P02751	HSP90B1	P14625
IL6	P05231	KNG1	P01042	MSLN	Q13421
PLG	P00747	SERPINA1	P01009	SERPINC1	P01008

21. Post-translational protein phosphorylation (R-HSA-8957275)

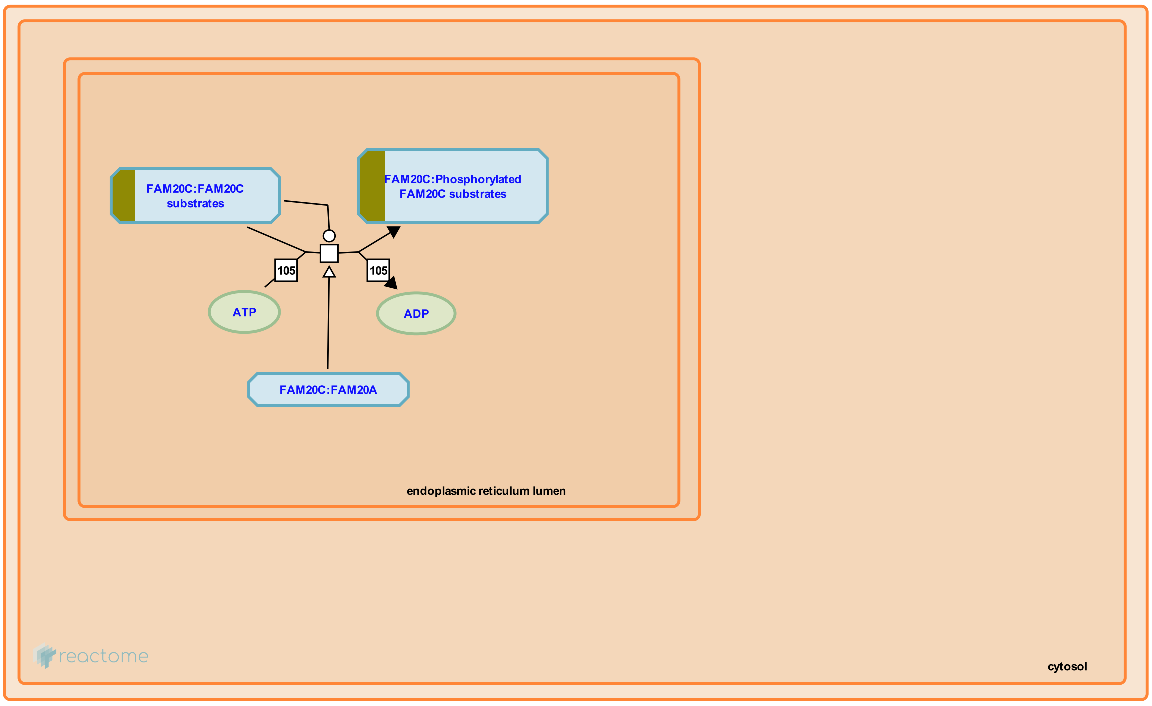

Secretory pathway kinases phosphorylate a diverse array of substrates involved in many physiological processes.

References

Sreelatha A, Kinch LN & Tagliabracci VS (2015). The secretory pathway kinases. Biochim. Biophys. Acta, 1854, 1687-93.

Edit history

Date	Action	Author
2016-12-08	Authored	Jupe S
2017-01-23	Reviewed	Wiley SE
2017-01-24	Edited	Jupe S
2017-01-24	Created	Jupe S
2021-05-31	Modified	Shorser S

Entities found in this pathway (16)

Input	UniProt Id	Input	UniProt Id	Input	UniProt Id
AHSG	P02765	APOB	P04114	APOE	P02649
C3	P01024	C4A	P0C0L4	C4B	P0C0L4
CP	P00450	FGA	P02671	FGG	P02679
FN1	P02751	HSP90B1	P14625	IL6	P05231
KNG1	P01042	MSLN	Q13421	SERPINA1	P01009
SERPINC1	P01008				

22. Activation of C3 and C5 (R-HSA-174577)

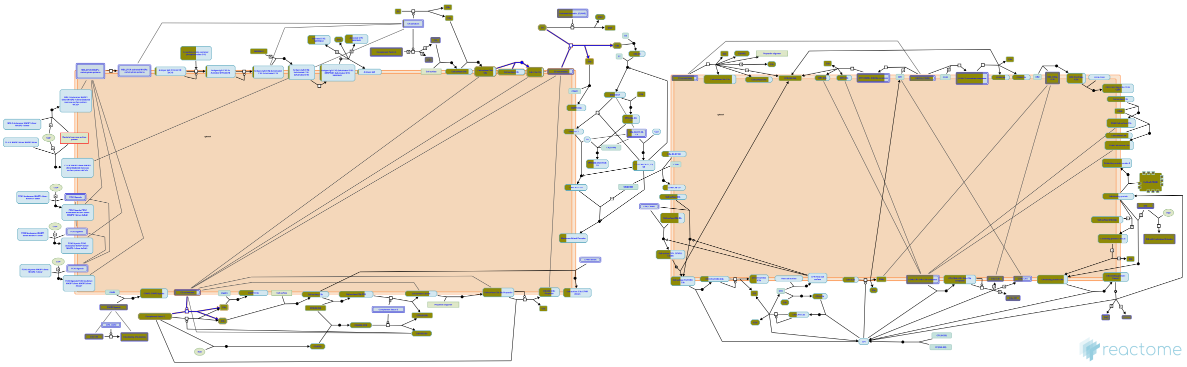

Cellular compartments: extracellular region, plasma membrane.

The 3 pathways of complement activation converge on the cleavage of C3 by C3 convertases. C3 convertase cleaves C3 into C3a and C3b - a central step of complement activation. C3a remains in the fluid phase and acts as an anaphylatoxin, whereas C3b can form additional C3 convertases hastening the production of C3b. Besides, C3b binds to C3 convertases to form C5 convertase, which can act as an opsonin, or is degraded into fragments which cannot form an active convertase.

References

Lesavre PH, Hugli TE, Esser AF & Muller-Eberhard HJ (1979). The alternative pathway C3/C5 convertase: chemical basis of factor B activation. J Immunol, 123, 529-34.

Fujita T, Matsushita M & Endo Y (2004). The lectin-complement pathway--its role in innate immunity and evolution. Immunol Rev, 198, 185-202.

Edit history

Date	Action	Author
2004-08-04	Authored	de Bono B
2006-02-20	Created	de Bono B
2006-07-04	Reviewed	D'Eustachio P
2010-11-17	Edited	Jupe S
2021-05-31	Modified	Shorser S

Entities found in this pathway (6)

Input	UniProt Id	Input	UniProt Id	Input	UniProt Id
C2	P06681	C3	P01024	C4A	P0C0L4, P0C0L5
C4B	P0C0L4, P0C0L5	C5	P01031	CFB	P00751

23. Regulation of Complement cascade (R-HSA-977606)

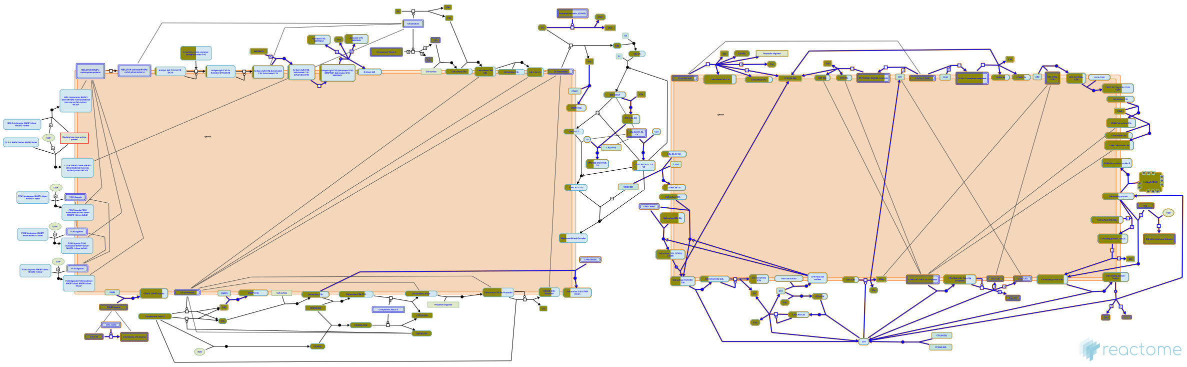

Cellular compartments: plasma membrane, extracellular region.

Two inherent features of complement activation make its regulation very important:

1. There is an inherent positive feedback loop because the product of C3 activation forms part of an enzyme that causes more C3 activation.

2. There is continuous low-level activation of the alternative pathway (see Spontaneous hydrolysis of C3 thioester).

Complement cascade activation is regulated by a family of related proteins termed the regulators of complement activation (RCA). These are expressed on healthy host cells. Most pathogens do not express RCA proteins on their surface, but many have found ways to evade the complement system by stably binding the RCA that circulates in human plasma (Lambris et al. 2008); trapping RCA is by far the most widely employed strategy for avoiding the complement response. RCA recruitment is common in bacteria such as E. coli and streptococci (Kraiczy & Wurzner 2006) and has also been described for viruses, fungi and parasites. RCA deposition and the complement system also have an important role in tissue homeostasis, clearing dead cells and debris, and preventing damage from oxidative stress (Weismann et al. 2011).

RCA proteins control complement activation in two different ways; by promoting the irreversible dissociation (decay acceleration) of complement convertases and by acting as cofactors for Complement factor I (CFI)-mediated cleavage of C3b and C4b.

Decay accelerating factor (DAF, CD55), Complement factor H (FH), Membrane Cofactor Protein (MCP) and Complement receptor 1 (CR1) are composed of arrays of tandem globular domains termed CCPs (complement control protein repeats) or SCRs (short consensus repeats). CR1, MCP and FH are cofactors for the CFI-mediated cleavage of C3b, generating iC3b. CR1 and MCP are also cofactors for C4b cleavage.

C4BP is an additional cofactor for the CFI-mediated cleavage of C4b.

References

Gasque P (2004). Complement: a unique innate immune sensor for danger signals. Mol Immunol, 41, 1089-98.

Zipfel PF & Skerka C (2009). Complement regulators and inhibitory proteins. Nat Rev Immunol, 9, 729-40.

Ricklin D, Hajishengallis G, Yang K & Lambris JD (2010). Complement: a key system for immune surveillance and homeostasis. Nat Immunol, 11, 785-97.

The Complement System. Retrieved from http://users.rcn.com/jkimball.ma.ultranet/BiologyPages/C/Complement.html

Edit history

Date	Action	Author
2010-10-20	Created	Jupe S
2010-10-26	Authored	Jupe S
2010-11-01	Edited	Jupe S
2012-02-13	Reviewed	Fraczek LA, Bradley DT
2021-05-31	Modified	Shorser S

Entities found in this pathway (14)

Input	UniProt Id	Input	UniProt Id	Input	UniProt Id
C1QA	P02745	C1QB	P02746, P02747	C2	P06681
C3	P01024	C4A	P0C0L4, P0C0L5	C4B	P0C0L4, P0C0L5
C4BPA	P04003, P15529	C5	P01031	CFB	P00751
F2	P00734	IGLC1	P0CG04	PROS1	P07225
SERPING1	P05155	VTN	P04004		

24. Neutrophil degranulation (R-HSA-6798695)

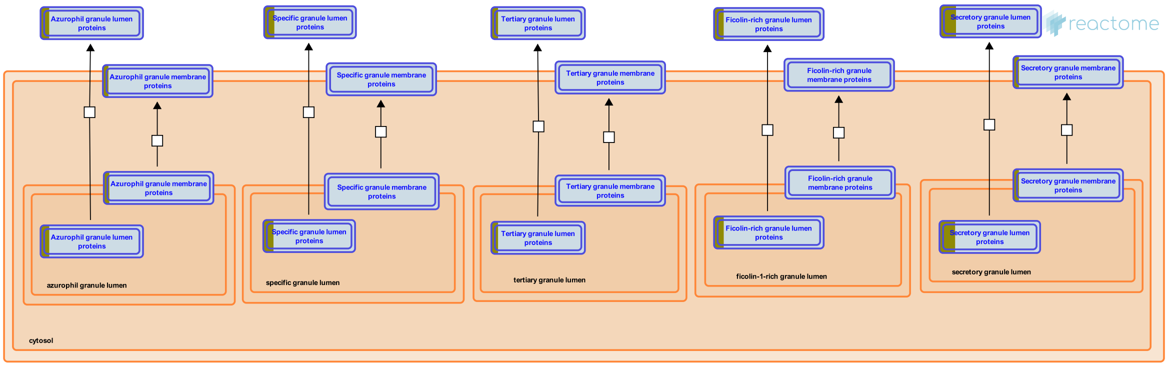

Neutrophils are the most abundant leukocytes (white blood cells), indispensable in defending the body against invading microorganisms. In response to infection, neutrophils leave the circulation and migrate towards the inflammatory focus. They contain several subsets of granules that are mobilized to fuse with the cell membrane or phagosomal membrane, resulting in the exocytosis or exposure of membrane proteins. Traditionally, neutrophil granule constituents are described as antimicrobial or proteolytic, but granules also introduce membrane proteins to the cell surface, changing how the neutrophil responds to its environment (Borregaard et al. 2007). Primed neutrophils actively secrete cytokines and other inflammatory mediators and can present antigens via MHC II, stimulating T-cells (Wright et al. 2010).

Granules form during neutrophil differentiation. Granule subtypes can be distinguished by their content but overlap in structure and composition. The differences are believed to be a consequence of changing protein expression and differential timing of granule formation during the terminal processes of neutrophil differentiation, rather than sorting (Le Cabec et al. 1996).

The classical granule subsets are Azurophil or primary granules (AG), secondary granules (SG) and gelatinase granules (GG). Neutrophils also contain exocytosable storage cell organelles, storage vesicles (SV), formed by endocytosis they contain many cell-surface markers and extracellular, plasma proteins (Borregaard et al. 1992). Ficolin-1-rich granules (FG) are like GGs highly exocytosable but gelatinase-poor (Rorvig et al. 2009).

References

Wright HL, Moots RJ, Bucknall RC & Edwards SW (2010). Neutrophil function in inflammation and inflammatory diseases. Rheumatology (Oxford), 49, 1618-31.

Borregaard N, Sørensen OE & Theilgaard-Mönch K (2007). Neutrophil granules: a library of innate immunity proteins. Trends Immunol., 28, 340-5.

Rørvig S, Østergaard O, Heegaard NH & Borregaard N (2013). Proteome profiling of human neutrophil granule subsets, secretory vesicles, and cell membrane: correlation with transcriptome profiling of neutrophil precursors. J. Leukoc. Biol., 94, 711-21.

Borregaard N, Kjeldsen L, Rygaard K, Bastholm L, Nielsen MH, Sengeløv H, ... Johnsen AH (1992). Stimulus-dependent secretion of plasma proteins from human neutrophils. J. Clin. Invest., 90, 86-96.

Le Cabec V, Cowland JB, Calafat J & Borregaard N (1996). Targeting of proteins to granule subsets is determined by timing and not by sorting: The specific granule protein NGAL is localized to azurophil granules when expressed in HL-60 cells. Proc. Natl. Acad. Sci. U.S.A., 93, 6454-7.

Edit history

Date	Action	Author
2015-09-21	Authored	Jupe S
2015-09-21	Created	Jupe S
2016-06-13	Edited	Jupe S
2016-06-13	Reviewed	Heegaard N
2021-05-22	Modified	Shorser S

Entities found in this pathway (29)

Input	UniProt Id	Input	UniProt Id	Input	UniProt Id
A1BG	P04217	AGL	P35573	AHSG	P02765
ANXA2	P07355	B2M	P61769	C3	P01024
CAP1	Q01518	CCT2	P78371	COTL1	Q14019
CXCL1	P09341	CXCL8	P09341	EEF2	P13639
GPI	P06744	GSTP1	P09211	HLA-B	P01889
HLA-C	P10321	HMGB1	P09429	HP	P00738
HSP90AA1	P07900	HUWE1	Q7Z6Z7	LCN2	P80188
LTF	P02788	ORM2	P19652	PSAP	P07602
S100A11	P31949	S100A9	P06702	SERPINA1	P01009, P01011
VAT1	Q99536	VCL	P18206		

25. Hemostasis (R-HSA-109582)

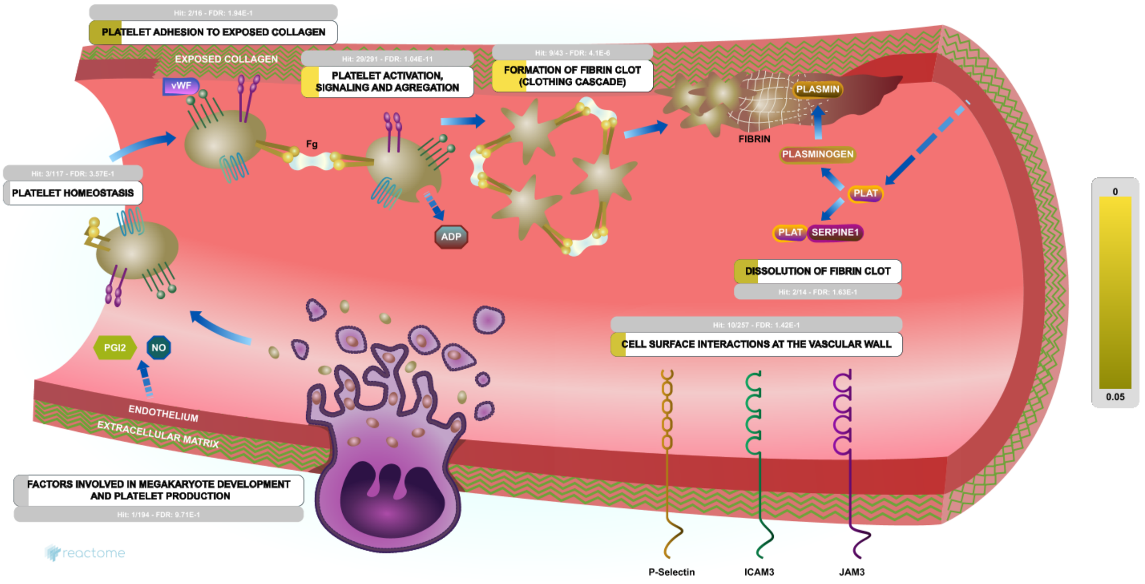

Hemostasis is a physiological response that culminates in the arrest of bleeding from an injured vessel. Under normal conditions the vascular endothelium supports vasodilation, inhibits platelet adhesion and activation, suppresses coagulation, enhances fibrin cleavage and is anti-inflammatory in character. Under acute vascular trauma, vasoconstrictor mechanisms predominate and the endothelium becomes prothrombotic, procoagulatory and proinflammatory in nature. This is achieved by a reduction of endothelial dilating agents: adenosine, NO and prostacyclin; and by the direct action of ADP, serotonin and thromboxane on vascular smooth muscle cells to elicit their contraction (Becker et al. 2000).

The chief trigger for the change in endothelial function that leads to the formation of a haemostatic thrombus is the loss of the endothelial cell barrier between blood and extracellular matrix components (Ruggeri 2002). Circulating platelets identify and discriminate areas of endothelial lesions; here, they adhere to the exposed sub endothelium. Their interaction with the various thrombogenic substrates and locally generated or released agonists results in platelet activation. This process is described as possessing two stages, firstly, adhesion - the initial tethering to a surface, and secondly aggregation - the platelet-platelet cohesion (Savage & Cattaneo et al. 2001).

Three mechansism contribute to the loss of blood following vessel injury. The vessel constricts, reducing the loss of blood. Platelets adhere to the site of injury, become activated and aggregate with fibrinogen into a soft plug that limits blood loss, a process termed primary hemostasis. Proteins and small molecules are released from granules by activated platelets, stimulating the plug formation process. Fibrinogen from plasma forms bridges between activated platelets. These events initiate the clotting cascade (secondary hemostasis). Negatively-charged phospholipids exposed at the site of injury and on activated platelets interact with tissue factor, leading to a cascade of reactions that culminates with the formation of an insoluble fibrin clot.

References

Colman R, Marder V, Clowes A, George J & Goldhaber S (2006). *Hemostasis and Thrombosis: Basic Principles and Clinical Practice* .

Allford S & Machin S (2004). *Haemostasis* , 200a - 200d.

Edit history

Date	Action	Author
2004-01-22	Authored	Farndale R, Pace NP, D'Eustachio P, de Bono B
2004-01-22	Created	Farndale R, Pace NP, D'Eustachio P, de Bono B
2021-05-18	Edited	Joshi-Tope G
2021-05-18	Reviewed	Brummel K, Stafford DW, Rush MG
2021-05-22	Modified	Shorser S

Entities found in this pathway (35)

Input	UniProt Id	Input	UniProt Id	Input	UniProt Id
A1BG	P04217	A2M	P01023	AHSG	P02765
ANXA2	P07355	APOB	P04114	CAP1	P47755, Q01518
CLEC3B	P05452	COL1A1	P02452	COL1A2	P08123
F2	P00734	F9	P00740	FGA	P02671
FGG	P02679	FLNA	P21333	FN1	P02751
GNAI2	P04899	GNAS	P63092, Q5JWF2	HGF	P14210
HSPA5	P11021	IGLC1	P0CG04	KNG1	P01042
LGALS3BP	Q08380	ORM2	P19652	PDGFB	P01127
PLG	P00747	PROS1	P07225	PSAP	P07602
SDC1	P18827	SERPINA1	P01009, P01011	SERPINC1	P01008
SERPING1	P05155	TGFB1	P01137	TSTD1	P78310
VCL	P18206	VEGFA	P15692, P49765		

6. Identifiers found

Below is a list of the input identifiers that have been found or mapped to an equivalent element in Reactome, classified by resource.

Entities (137)

Input	UniProt Id	Input	UniProt Id	Input	UniProt Id
A1BG	P04217	A2M	P01023	ACTR3	P61158
ADM	P35318	AGL	P35573	AHSG	P02765
ANXA1	P04083	ANXA2	P07355	APOB	P04114
APOC3	P02656	APOE	P02649	ARF1	P84077
AZGP1	P25311	B2M	P61769	C1QA	P02745
C1QB	P02746, P02747	C2	P06681	C3	P01024
C4A	P0C0L4, P0C0L5	C4B	P0C0L4, P0C0L5	C4BPA	P04003, P15529
C5	P01031	CALR	P27797	CAP1	Q01518
CCL2	P13500	CCL3L3	P16619	CCL4	O00626, P13236
CCL4L2	Q8NHW4	CCL5	P13501	CCT2	P78371
CDH1	Q9UM11	CFB	P00751	CLEC3B	P05452
CLTC	Q00610, Q00610-1	COL18A1	P39060	COL1A1	P02452
COL1A2	P08123	COL4A4	P53420	COTL1	Q14019
CP	P00450	CSF2	P04141	CSF3	P09919
CXCL1	P09341	CXCL2	P19875	CXCL8	P09341, P10145
DCN	P07585	DFFA	O00273	DPYSL3	Q14195
EEF1D	P29692	EEF2	P13639	EZR	P15311
F2	P00734	F9	P00740	FBLN1	P23142
FGA	P02671	FGF18	O76093	FGF7	P21781
FGG	P02679	FLNA	P21333	FN1	P02751
GARS	P41250	GC	P38435	GNAI2	P04899
GNAS	P63092, Q5JWF2	GPI	P06744	GSTA1	P08263
GSTP1	P09211	HBEGF	Q99075	HGF	P14210
HLA-B	P01889, P03989, P10319, P18463, P18464, P18465, P30460, P30461, P30462, P30464, P30466, P30475, P30479, P30480, P30481, P30483, P30484, P30485, P30486, P30487, P30488, P30490, P30491, P30492, P30493, P30495, P30498, P30685, Q04826, Q29718, Q29836, Q29940, Q31610, Q31612, Q95365	HLA-C	P04222, P10321, P30499, P30501, P30504, P30505, P30508, P30510, Q07000, Q29865, Q29960, Q29963, Q95604, Q9TNN7	HLA-G	P17693
HMGB1	P09429	HNRNPA2B1	P22626	HP	P00738
HSP90AA1	P07900	HSP90B1	P14625	HSPA5	P11021
HSPB1	P04792	HSPH1	Q92598	HUWE1	Q7Z6Z7, Q8IYU2
IFNG	P01579	IGLC1	P0CG04	IL10	P22301
IL1B	P01583, P01584	IL3	P08700	IL4	P05112
IL6	P05231	IL7	P13232	KNG1	P01042
LCN2	P80188	LGALS3BP	Q08380	LTA	P01374
LTF	P02788	MAP2K6	P52564	MAPT	P10636
MDK	P21741	MMP7	P09237	MRI1	Q9BV20

Input	UniProt Id	Input	UniProt Id	Input	UniProt Id
MSLN	Q13421	NAXE	Q8NCW5	NRCAM	Q92823
NTF3	P20783	ORM2	P19652	OSM	P13725
OVGP1	Q12889	PDGFB	P01127	PDIA3	P30101
PFKP	Q01813	PLG	P00747	PROS1	P07225
PSAP	P07602	PSMA7	O14818, Q8TAA3	PTGR2	Q8N8N7
QDPR	P09417	RPL3	P39023, Q92901	RPS19	P39019
S100A11	P31949	S100A9	P06702	SDC1	P18827
SERPINA1	P01009, P01011	SERPINC1	P01008	SERPING1	P05155
SHMT1	P34896	SNCA	P37840	TGFB1	P01137
THY1	P04216	TNF	P01375	TP53AIP1	Q9HCN2
TSTD1	P78310	UBA3	Q8TBC4	UGDH	O60701
VAT1	Q99536	VCL	P18206	VEGFA	P15692, P49765
VTN	P04004	WNT10A	Q9GZT5		

Input	Ensembl Id	Input	Ensembl Id	Input	Ensembl Id
ANXA1	ENSG00000135046	ANXA2	ENSG00000182718	APOE	ENSG00000130203
ARF1	ENSG00000143761	B2M	ENSG00000166710	CALR	ENSG00000179218
CCL2	ENSG00000108691	CCL3L3	ENSG00000277768	CCL4	ENSG00000275302
CCL5	ENSG00000271503	COL1A1	ENSG00000108821	COL1A2	ENSG00000164692
CSF2	ENSG00000164400	CSF3	ENSG00000108342	CXCL1	ENSG00000163739
CXCL2	ENSG00000081041	CXCL8	ENSG00000169429	FN1	ENSG00000115414
HGF	ENSG00000019991	HLA-B	ENSG00000234745	HLA-C	ENSG00000204525
HLA-G	ENSG00000204632	HNRNPA2B1	ENSG00000122566	HSP90AA1	ENSG00000080824
HSP90B1	ENSG00000166598	HSPA5	ENSG00000044574	HSPB1	ENSG00000106211
HSPH1	ENSG00000120694	IFNG	ENSG00000111537	IL10	ENSG00000136634
IL1B	ENSG00000125538	IL3	ENSG00000164399	IL6	ENSG00000136244
LCN2	ENSG00000148346	OSM	ENSG00000099985	PSAP	ENSG00000122852, ENSG00000185303
TGFB1	ENSG00000105329	TNF	ENSG00000232810	TP53AIP1	ENSG00000120471
VEGFA	ENSG00000112715				

7. Identifiers not found

These 8 identifiers were not found neither mapped to any entity in Reactome.

ANXA2P2	IGLL5	IPO9	PAEP	SAA4	SFRP4	SHBG	TGM2
